# ChromInSight: Revealing DNA Double‐Strand Breaks Through Chromatin Structural Insights With an Interpretable Graph Neural Network Framework

**DOI:** 10.1002/advs.202504571

**Published:** 2025-06-30

**Authors:** Kang Xu, Zongyuan Yu, Canzhuang Sun, Conglin Gou, Jiangyue Zhu, Jun Wang, Xiaochen Bo, Guoxian Yu, Hao Li, Hebing Chen

**Affiliations:** ^1^ Academy of Military Medical Sciences; ^2^ School of Software Shandong University China

**Keywords:** DSB, Graph Contrastive Learning, 3D Chromatin Spatial Conformation, Benchmark

## Abstract

DNA double‐strand breaks (DSBs) represent one of the most severe forms of genomic damage. Although substantial progress has been made in elucidating general patterns associated with DSBs, the influence of 3D chromatin structure on DSB formation remains underexplored, particularly concerning its spatial configuration. Here, the ChromInSight framework is introduced. Using standardized datasets,Hi‐DSB is developed and deployed in ChromInSight, a genome‐wide DSB prediction model based on graph contrastive learning (GCL), and applied advanced interpretability techniques to identify DSB‐associated genomic patterns. The findings reveal that the spatial cluster‐scene between hub nodes and DSB sites is predominantly shaped by the 3D conformation of chromatin, rather than by linear genomic distance. This phenomenon is validated at both the Loop and topologically associating domain (TAD) levels and proposed a “spatial isolation – damage containment” hypothesis, which illustrates the genome strategy for managing damage. These findings support the role of 3D genome architecture in genomic instability. Consequently, the framework provides a powerful tool for investigating the intricate relationship between chromatin structure and genomic stability.

## Graphical abstract

DSBs are the most critical genomic lesions. Using the ChromInSight framework, It is revealed that 3D chromatin structure, rather than linear distance, shapes cluster‐scene between hub‐Nodes and DSB loci. The “spatial isolation–damage containment” hypothesis is proposed to illustrate how the genome responds to genomic damage and offer a powerful tool for studying genome stability.

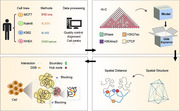

## 1 Introduction

DeoxyriboNucleic Acid (DNA) double‐strand breaks (DSBs) involve the simultaneous severing of both strands of the DNA double helix, a type of damage that can be precipitated by a variety of factors, including ionizing radiation and certain chemical agents.^[^ ^1^, ^2^ ^]^ As one of the most severe types of damage, DSBs pose dual threats to genomic integrity through direct gene inactivation and chromosomal rearrangement cascades, processes intrinsically linked to oncogenic transformation.^[^ ^3^, ^4^ ^]^ Given the severe implications of DSBs for genomic integrity,^[^ ^5^ ^]^ establishing and improving DSB detection techniques is crucial for understanding DSB events.^[^ ^6^ ^]^ Recent technological breakthroughs exemplified by high‐resolution DSB detection methods (BLESS,^[^ ^7^ ^]^ BLISS,^[^ ^8^ ^]^ DSBCapture,^[^ ^6^ ^]^ END‐seq^[^ ^9^ ^]^) have revolutionized our capacity to profile DSB landscapes at single‐base‐pair resolution across entire genomes.

The integration of multi‐omics data such as Hi‐C,^[^ ^10^ ^]^ DNase‐seq,^[^ ^11^ ^]^ ChIP‐seq,^[^ ^12^ ^]^ and bioinformatics offers a comprehensive evident DSBs landscape. Emerging evidence demonstrates that DSBs exhibit pronounced genomic stratification,^[^ ^13^, ^14^, ^15^ ^]^ with preferential accumulation in transcriptional regulatory elements including gene promoters^[^ ^16^, ^17^ ^]^ and active enhancers^[^ ^18^, ^19^ ^]^ marked by H3K27ac/H3K4me3 histone modifications.^[^ ^20^, ^21^, ^22^ ^]^ Sequence‐level preferences are evident through AP‐1 transcription factor motif enrichment at breakage loci.^[^ ^20^ ^]^ Crucially, DSB hotspots colocalize with 3D genome organizational features, showing significant overlap with loop anchors and topologically associating domain (TAD) boundaries,^[^ ^5^, ^23^, ^24^ ^]^ suggesting chromatin conformation exerts hierarchical control over break susceptibility.

The 3D organization of chromatin participates in the DNA damage response^[^ ^14^ ^]^ TADs, as basic units of chromatin spatial organization,^[^ ^25^, ^26^, ^27^ ^]^ exhibit elevated intra‐domain chromatin interactions bounded by insulated regulatory elements.^[^ ^28^ ^]^ This architectural partitioning contributes to the regulation of gene expression and the specificity of enhancer–promoter interactions^[^ ^29^ ^]^ and the biophysical constraints shaping DSB repair microenvironments.^[^ ^30^ ^]^ Current models posit that cohesin‐mediated loop extrusion dynamically assembles chromatin repair platforms within TAD confines.^[^ ^30^ ^]^ Following DSB generation, immediate phosphorylation of histone H2AX (γH2AX) at damage sites initiates a cascade wherein loop extrusion machinery propagates this modification radially, generating a chromatin state optimized for repair factor assembly.^[^ ^30^ ^]^ Parallel studies demonstrate that repair factors such as 53BP1 and RIF1 can form functionally autonomous modules that stabilize the topological architecture at the break site and help preserve the epigenetic landscape of the damaged region.^[^ ^31^ ^]^

However, the current understanding of the relationship between TADs and DSBs remains poorly integrated. Accumulating evidence supports the role of TAD‐restricted loop extrusion in scaffolding DNA repair platforms, yet comprehensive maps delineating TAD‐specific protection mechanisms or vulnerability profiles remain absent. Although 3D chromatin architecture offers a structural foundation for investigating DNA damage responses, the potential of Hi‐C data in identifying DSB sites has not been fully exploited.^[^ ^32^ ^]^ Moreover, the causal relationship between intra‐TAD chromatin compaction states and DSB susceptibility represents a blind spot. This knowledge gap hinders our ability to decipher how chromatin architecture governs DSB distribution and repair outcomes within the complex 3D organization of the genome. A deeper understanding of these relationships is essential for uncovering the broader impact of chromatin conformation on genome stability.

Despite significant advances in multi‐omics techniques and high‐throughput DSB sequencing, the high cost and technical challenges limit broader application.^[^ ^20^ ^]^ Artificial intelligence integration with multi‐omics data emerges as a strategic pathway to overcome these limitations.^[^ ^32^ ^]^ Current efforts in this domain, however, remain nascent. Established methodologies fall into two classes: classical machine learning algorithms including Random Forests,^[^ ^20^, ^32^ ^]^ and advanced deep learning architectures like DSB‐GNN.^[^ ^33^ ^]^ Each category brings distinct advantages and faces unique challenges.

**Machine learning model**: In 2018, Raphael Mourad et al.^[^ ^20^ ^]^ applied a random forest model based on a balanced dataset to predict DSBs. However, the intrinsic genomic imbalance between DSB and non‐DSB regions persists as a fundamental constraint on model generalizability, necessitating rigorous validation under natural data distributions.^[^ ^33^ ^]^ Subsequent work by Ballinger et al.^[^ ^32^ ^]^ in 2019 through systematic integration of Hi‐C, CTCF, and DNase signals across 50 kb genomic windows, with Pearson correlation coefficients serving as the primary metric for identifying DSB loci. Although demonstrating predictive utility, the absence of standardized classification metrics particularly the area under the receiver operating characteristic (AUROC) and area under the precision‐recall curve (AUPRC) restricts mechanistic interpretation and cross‐study benchmarking, thereby impeding comparative analyses with alternative methodologies.

**Deep learning model**: Sun et al.^[^ ^33^ ^]^ introduced a graph neural network (DSB‐GNN) in 2023 to investigate DSB interactions with 3D chromatin architecture in normal human epidermal keratinocytes (NHEK), revealing “bottleneck” genomic regions critical for chromatin integrity. Persistent limitations constrain broader applicability. First, although DSB‐GNN performed well in a single normal cell line, its ability to detect similar bottleneck regions in cancer cells has not been tested. Second, the model focuses on subgraphs formed by the top 10 edges among first‐order neighbors of each node, potentially overlooking contributions from more distant but still relevant genomic regions. Finally, DSB‐GNN primarily examines the relationship between chromatin structure and DSBs at the 2D network level, lacking a 3D spatial perspective on DSB phenomena.

In response to these requirements and to gain a comprehensive understanding of DSB behavior in the context of broader interactions and complex 3D conformations, we propose ChromInSight — a genomic panoramic analysis framework that consists of three modules.

**1. DSB Dataset Module**: We curated seven DSB datasets that underwent quality‐controlled alignment and peak calling, forming a multi‐omics reference atlas for systematic DSB classification. This resource enables cross‐comparative annotation of DSB site features while establishing quality benchmarks for genome‐wide DSB detection.

**2. DSB Prediction Module**: We developed Hi‐DSB, a genome‐wide DSB prediction module based on graph contrastive learning. Hi‐DSB constructs genomic graphs using Hi‐C data by partitioning the genome into bins, with each bin represented as a node and bin‐bin interactions as edges. Epigenomic profiles are incorporated as node features. This approach integrates both chromatin conformation and regulatory information, offering a comprehensive view of DSB formation. To enhance model interpretability, we employed GNNExplainer, a graph neural network interpretability method, to identify key genomic patterns associated with DSBs. This enables Hi‐DSB not only achieves in predict genome‐wide DSBs, but also to offer mechanistic insights into how chromatin organization influences DNA damage susceptibility.

**3. Feature Interpretation Module**: This module, from the perspective of 3D chromatin spatial structure, reveals that the cluster‐scene between hub nodes and DSB sites is primarily influenced by the spatial conformation of chromatin rather than linear genomic distances. Additionally, we identified genes impacting breast cancer patient survival from the hub nodes recognized by Hi‐DSB, providing new insights into the interplay between chromatin structure, genomic stability, and cancer progression.

## 2 Results

### 2.1 ChromInSight Overview

**Figure 1:**
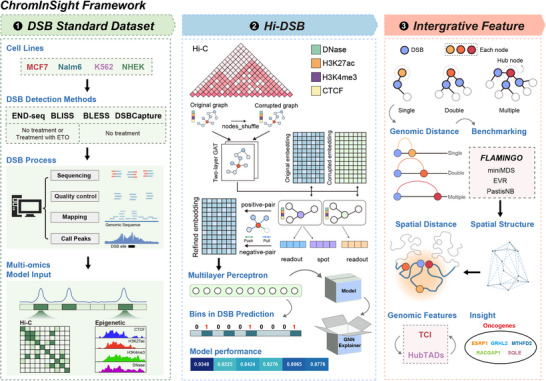
Schematic flow chart of ChromInSight. We introduce the ChromInSight framework, Our framework consists of three key modules. ①DSB Standard Dataset Construction Module: This module is designed to construct a standardized dataset for the prediction and interpretation of DSB events. ②Whole‐Genome DSB Prediction Module (Hi‐DSB): Leveraging a graph contrastive learning approach, this module develops the Hi‐DSB model to predict DSBs across the entire genome. ③Feature Integration and Analysis Module: This module aims to uncover patterns associated with genomic DSBs through integrated feature analysis.

ChromInSight is an ensemble framework for DSB studies and consists of three main components (**Figure** **1**). First, ChromInSight integrates a comprehensive collection and annotation of existing DSB data. Second, ChromInSight contains our novel Hi‐DSB approach (Experimental Section), which incorporates a graph contrastive learning‐based model and advanced interpretable techniques to capture genomic patterns associated with DSBs. Third, ChromInSight enables the discovery of local chromatin features linked to DSB occurrence, offering mechanistic insights into their formation.

In the DSB dataset module (Figure S1A; Table S1, Supporting Information; Table 1, Experimental Section), seven published DSB datasets were implemented, including two major DSB sources and four mainstream DSB detection sequencing technologies across four cell lines. Following standardized peak calling (Experimental Section), systematic benchmarking revealed key characteristics of DSB distributions. First, tumor cell lines (MCF7, K562, and Nalm6) exhibited distinct DSB genomic localization patterns (Figure S1,C, Supporting Information). Second, DSB peaks preferentially occupied distal intergenic regions, introns, and promoters (Figure S1D, Supporting Information), and showed reproducibility between DSBCapture and BLESS methods (Figure S2A–C, Supporting Information). Third, ETO‐induced DSBs demonstrated preferential vulnerability at endogenous DSB loci (Figure S2D,E, Supporting Information), while regulatory element colocalization^[^ ^16^, ^17^, ^19^ ^]^ (Figure S2F, S3C,D, Supporting Information) and enrichment at active chromatin regions—marked by H3K27ac,^[^ ^34^ ^]^ H3K4me3,^[^ ^34^ ^]^ DNase hypersensitive sites^[^ ^24^ ^]^ were consistently observed (Figure S2G, S3A,B, Supporting Information). Transcriptional analysis further linked DSB hotspots to promoters of highly expressed DSB‐associated genes^[^ ^17^ ^]^ (Figure S3E,F, Supporting Information). Collectively, these findings align with established DSB genomic distribution principles,^[^ ^18^, ^30^ ^]^ validating confidence in the quality of the dataset for downstream deep learning models.

**Figure 2:**
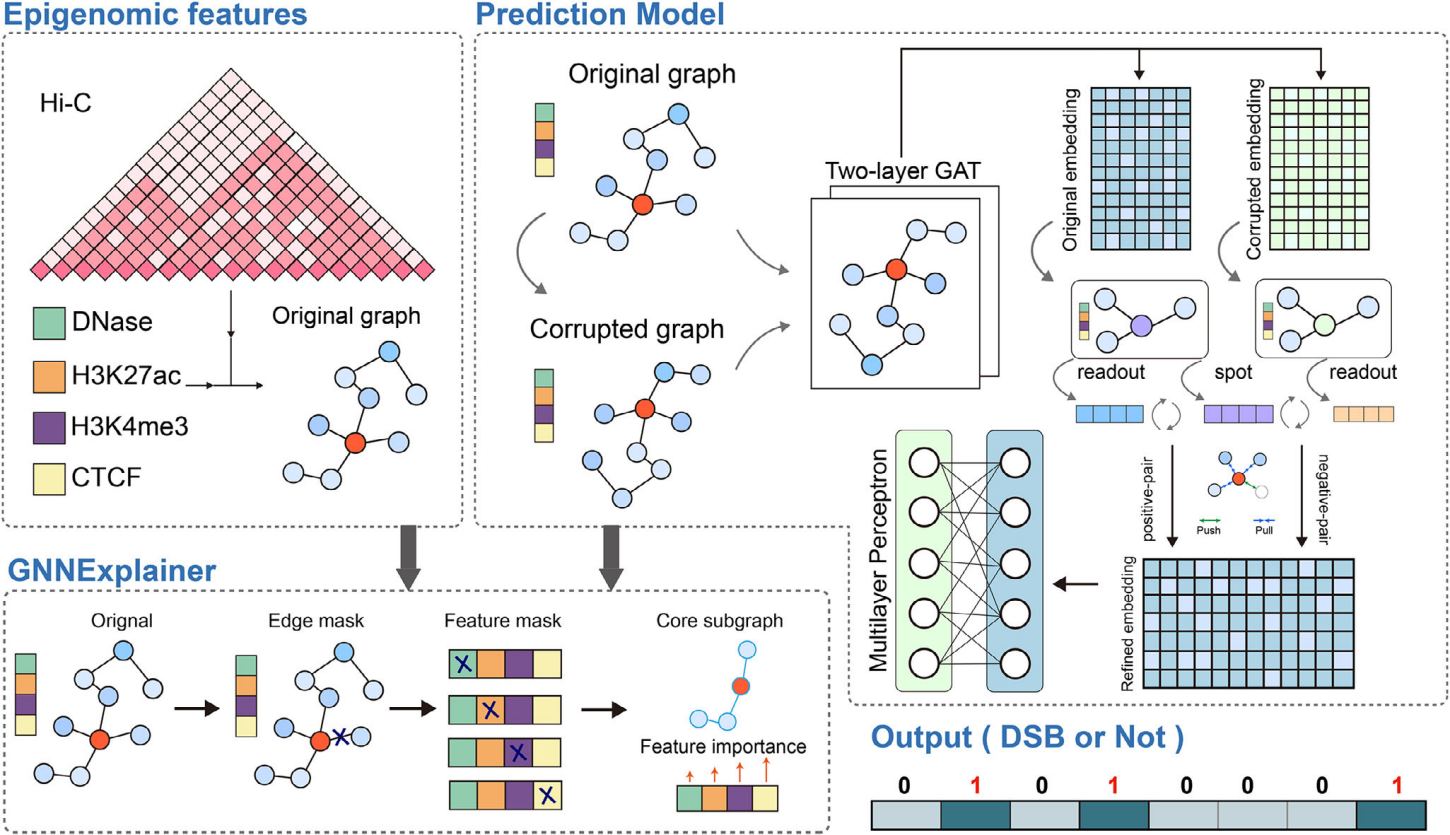
Overview of the Hi‐DSB framework. We constructed an interpretable DSB prediction model (Hi‐DSB) based on graph contrastive learning. Hi‐DSB utilized both epigenetic (CTCF, H3K27ac, H3K4me3, DNase) and Hi‐C features to construct a graph network. The model integrates Graph Attention Networks (GAT) with contrastive learning strategies, employing a multi‐level embedding generation and optimization process to achieve refined learning of node representations. The overall workflow of the model comprises four main components: input layer, embedding generation, contrastive learning, and output layer. Each module works in concert to perform efficient DSB prediction tasks. We further interpret the association between 3D chromatin structure and DSBs using GNNExplainer.

The DSB prediction module introduces Hi‐DSB (**Figure** **2**), an interpretable graph contrastive learning model that integrates 3D chromatin architecture with epigenetic features. Hi‐DSB constructs genome graphs by partitioning chromosomes into 10‐kb bins, where nodes represent genomic segments and edges encode Hi‐C‐derived interaction frequencies. Epigenomic profiles are mapped as nodal attributes, enabling simultaneous modeling of chromatin conformation and regulatory activity for DSB probability prediction (Experimental Section). To further interpret the relationship between 3D chromatin structure and DSBs, we applied GNNExplainer.^[^ ^35^ ^]^ GNNExplainer allowed us to dissect the contributions of specific nodes and edges in the graph, providing insights into how chromatin architecture influences DSB susceptibility.

In the feature interpretation module, we utilized GNNExplainer to identify the most critical nodes for predicting DSBs and classified these nodes based on the number of DSB sites they connect to into three categories: single‐node, double‐node, and multiple‐node (hub node). Our analysis revealed a notable clustering pattern between hub nodes and DSB sites (cluster‐scene). To explain this cluster scene, we approached it from the perspective of 3D chromatin spatial structure. Comparative evaluation of chromatin conformation reconstruction methods demonstrated these spatial clusters primarily arise from 3D chromatin organization rather than linear genomic proximity. We validated this cluster‐scene phenomenon at both the Loop and TAD levels, leading us to propose the “spatial isolation–damage sequestration” hypothesis, which describes a potential genomic strategy for managing DNA damage. These findings further support the idea that 3D genome architecture plays a critical role in modulating genomic instability.

### 2.2 Performance of Hi‐DSB in DSB Prediction

**Figure 3:**
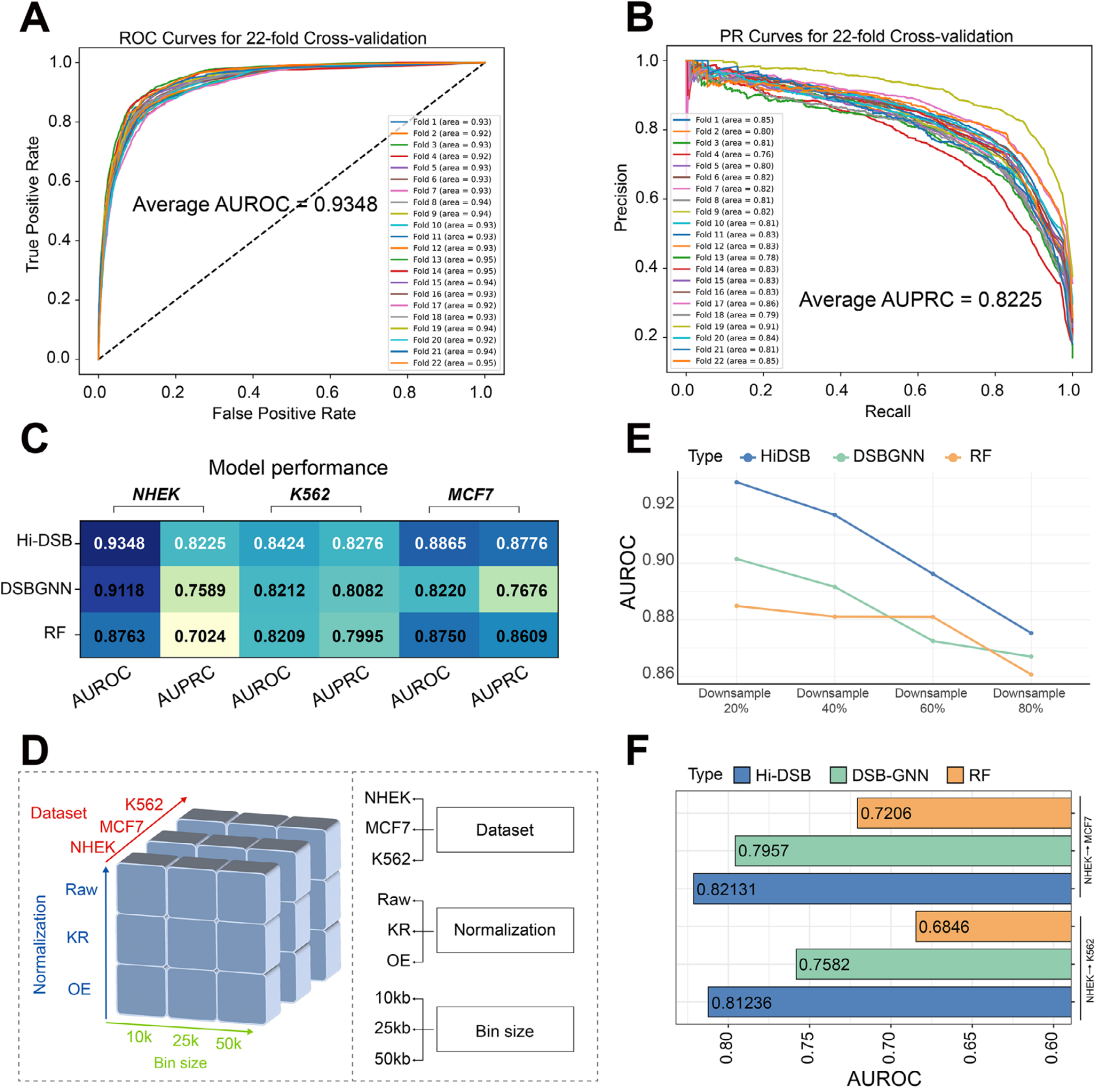
Hi‐DSB performance evaluation. (A,B) Performance Characteristics of Hi‐DSB inAUROC and AUPRC. To avoid overfitting, we conducted a 22‐fold cross‐validation, whereeach chromosome was sequentially used as an independent test set, while the remainingchromosomes formed the training set. This resulted in an Average AUROC of 0.934 and anAverage AUPRC of 0.822. (C) The average performance of three DSB prediction methodsacross different cell lines in 22‐fold cross‐validation is shown, with white highlighting thesuperior performance of Hi‐DSB across different cell lines. (D) Robustness evaluation of themodel across different Hi‐C scenarios. (E) Impact of Downsampling Hi‐C data on modelperformance. (F) Evaluate the model's ability to transfer from one cell line (NHEK) to twoadditional cell lines (K562 and MCF7).

ChromInSight's primary objective of predicting DSB events and identifying associated genomic features was achieved through the Hi‐DSB module. We therefore conducted analyses to evaluate Hi‐DSB's performance. Performance benchmarking in NHEK cells using a leave‐one‐out testing strategy^[^ ^36^ ^]^ (Experimental Section) yielded AUROC and AUPRC values of 0.934 and 0.822, respectively (**Figure** **3**A and 3B). We then compared Hi‐DSB with other existing methods and demonstrated that Hi‐DSB outperformed the state‐of‐the‐art method DSB‐GNN, as well as Random Forest‐based approaches,^[^ ^20^, ^32^, ^33^ ^]^ with consistent predictive accuracy across multiple cell lines (Figure 3C; Figure S5AB, Supporting Information).

Model robustness was quantified through 20 independent replicates with 95% confidence intervals, revealing Hi‐DSB's stability through higher AUROC/AUPRC scores and reduced variance (Figure S4A, Supporting Information). Error bar analysis (±1σ) confirmed minimal performance variability across iterations compared to benchmark methods (Figure S4B; Table S2A, Supporting Information). Statistical validation via DeLong's and McNemar's tests demonstrated significant improvements in predictive accuracy (p < 0.0001, DeLong test and McNemar test, Figure S4C,D; Table S2B,C, Supporting Information). These analyses establish Hi‐DSB as a statistically robust framework for genome‐scale DSB mapping.

For Hi‐C integrated models, systematic parameter sensitivity analyses revealed resolution‐dependent (10, 25 kb) performance variations (Figure S5C, D vs Figure 3C). We evaluated Hi‐DSB's adaptability across cell lines, Hi‐C resolutions, and normalization protocols compared to DSB‐GNN and random forest benchmarks (Figure 3D). Our results demonstrated that Hi‐DSB maintained competitive performance across these conditions (Tables S3,S4, Supporting Information). Progressive reads reduction simulations (20%–80% subsampling) demonstrated moderate accuracy declines across all models (Figure 3E), attributable to diminishing chromatin interaction signals. Despite data sparsity, Hi‐DSB preserved competitive AUROC/AUPRC metrics (Figure 3E; Figure S5E, Supporting Information), confirming robustness to sequencing depth fluctuations.

Hi‐DSB employs a graph contrastive learning architecture to integrate histone modification profiles with Hi‐C data, generating comprehensive chromatin interaction representations. Systematic ablation testing (Experimental Section) revealed that both model components and multimodal feature integration contributed substantially to prediction accuracy (Table S5, Supporting Information). Component‐specific evaluations confirmed the necessity of contrastive learning layers and chromatin features for optimal performance.

Cross‐cell generalizability was assessed through transfer learning experiments where Hi‐DSB trained on one cell line achieved superior predictive accuracy in two distinct cell lines compared to DSB‐GNN and random forest (Figure 3F; Figure S5F, Supporting Information). While performance attenuation occurred across transfers (attributable to cell‐type‐specific DSB,^[^ ^18^ ^]^ chromatin interaction patterns,^[^ ^37^ ^]^ and histone modification landscapes^[^ ^38^ ^]^), Hi‐DSB maintained higher AUPRC/AUROC than alternatives. This demonstrates its utility for constructing genome‐scale DSB maps in cell types with lacking direct DSB data.

External validation in HCT116 cells—an independent dataset absent from prior model benchmarking—confirmed Hi‐DSB's generalizability. Leave‐one‐out cross‐validation demonstrated superior performance (average AUROC 0.841) versus DSB‐GNN (0.813) and random forest (0.821) (Figure S6A–C, Supporting Information). Statistical validation through DeLong tests (p < 0.001) and McNemar tests (p < 0.001) confirmed Hi‐DSB's superiority across metrics (Table S6, Supporting Information). Multi‐resolution analysis (10, 25, 50 kb) and read depth simulations (20–100% subsampling), confirming competitive performance across different resolutions and various downsampled conditions (Figure S6D–F, Supporting Information).

In summary, the results showed that Hi‐DSB was a well‐performed DSB prediction module, and advanced in integrating Hi‐C data with other omics data.

### 2.3 The Cluster‐Scene between Hub Nodes and DSB Sites

**Figure 4:**
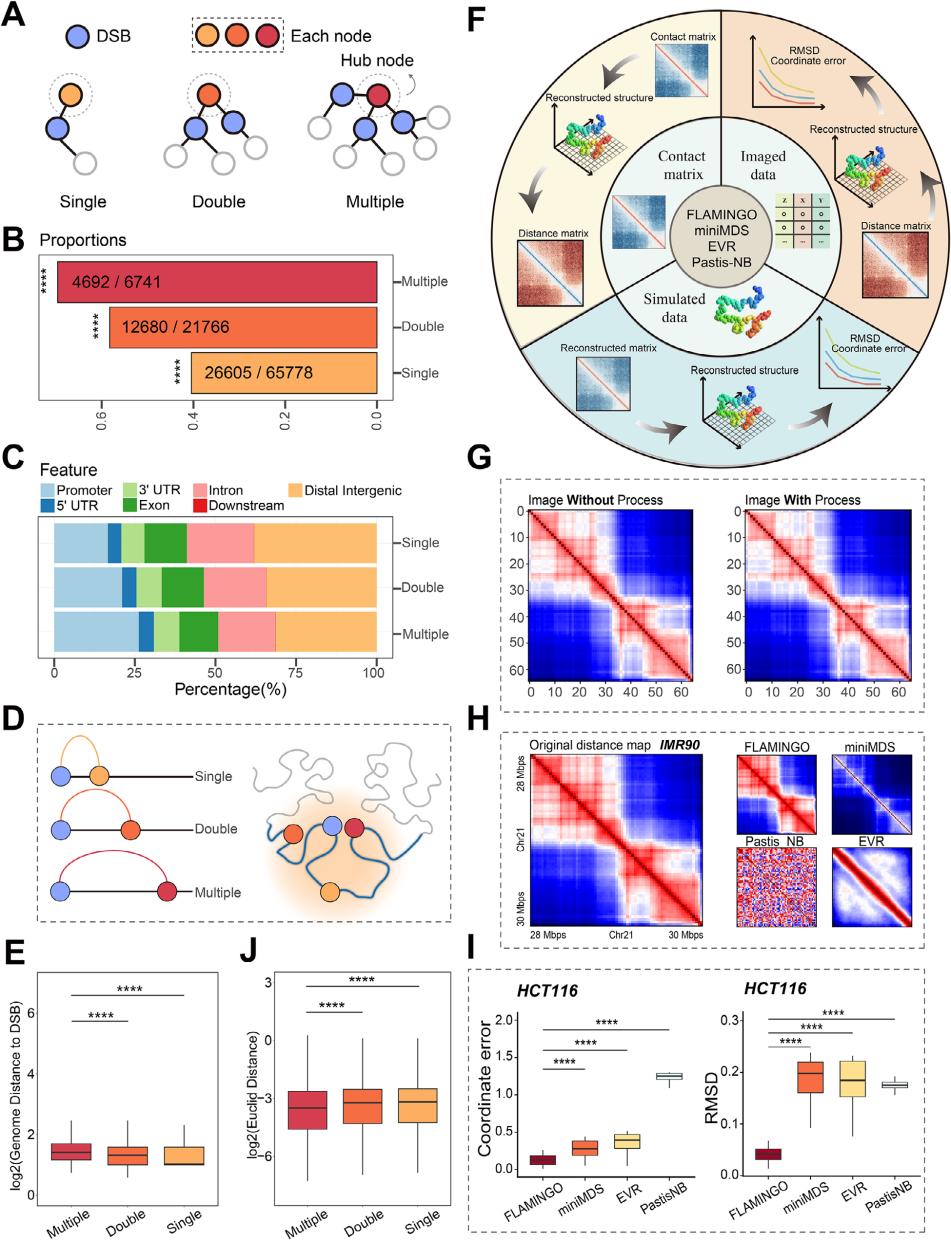
Hub nodes and DSB cluster‐scene. A) Schematic illustration of three patterns ofconnections between each node and DSB sites. Blue represents DSB sites, while the gradientfrom orange to red is used to distinguish nodes under different patterns. B) Probability of DSBoccurrence under three different patterns, ^**^ *p* < 0.01, ^***^ *p* < 0.001, ^****^ *p* < 0.0001, proportiontest. C) Genome‐wide distribution of nodes under three different patterns. D) Left panel:Illustrates the genomic linear distance between nodes and DSB sites under different patterns, serving as a schematic for Figure E. Right panel: Illustrates the spatial distance betweennodes and DSB sites under different patterns, serving as a schematic for Figure J. Blue represents DSB sites, while the gradient from orange to red is used to distinguish nodes underdifferent patterns. E) Genomic linear distance between nodes and DSB sites under differentpatterns. ^*^ *p* < 0.05, ^**^ *p* < 0.01, ^***^ *p* < 0.001, ^****^ *p* < 0.0001, Wilcox test. F) Schematicdiagram of benchmarking high‐resolution 3D chromatin reconstruction methods. G) Thecomparison between the processed (linear interpolation, centering, and F‐norm normalization)average distance map with the original average distance matrix (IMR90). H) Comparison ofcell average spatial distance matrices from different reconstruction methods and observedimage tracking data. I) RMSD and Coordinate error was employed to quantify the differencesbetween the reconstructed 3D chromatin structures using various methods andthe structures obtained through microscopy imaging. ^*^ *p* < 0.05, ^**^ *p* < 0.01, ^***^ *p* < 0.001, ^****^ *p* < 0.0001, Wilcox test. J) Genomic spatial distance (Euclid Distance) between nodes and DSBsites under different patterns. ^*^ *p* < 0.05, ^**^ *p* < 0.01, ^***^ *p* < 0.001, ^****^ *p* < 0.0001, Wilcox test.

To investigate potential factors influencing DNA fragility from a 3D genomic perspective, GNNExplainer was employed to identify chromatin interaction patterns contributing to lesion susceptibility. Critical chromatin interactions were mapped, with key nodes identified per genomic bin based on centrality metrics (Experimental Section). Three topological classes emerged (**Figure** **4**A): single‐pattern (one DSB node connection, n = 65778), double‐pattern (two connections, n = 21766), and multiple‐pattern (≥3 connections, n = 6741) termed hub nodes (Experimental Section). This classification framework also extended to non‐DSB nodes (Experimental Section) (Figure S7A, Supporting Information).

First, we analyzed the probability of DSB occurrence across different patterns. For cases involving a central DSB node (Experimental Section), we observed an increasing trend in DSB probability from the single‐pattern to the multiple‐pattern category (p < 0.0001, Proportion test, Figure 4B). In contrast, when the central node was a non‐DSB node (Experimental Section), the DSB probability decreased as the pattern transitioned from single‐pattern to multiple‐pattern (Figure S7B, Supporting Information). Nodes connected to more DSB sites were more likely to undergo breakage events, whereas nodes with fewer connections to DSB sites exhibited reduced susceptibility to DSBs (Figure S7C, Supporting Information). Notably, the trend was consistently observed in the K562 cell line as well (Figure S7D,E, Supporting Information). This phenomenon—reminiscent of the saying “birds of a feather flock together”—suggests that both the intrinsic properties of a node and its local network environment significantly influence the genomic stability of surrounding regions. We refer to this phenomenon as the cluster‐scene.

Genome‐wide mapping of interaction patterns revealed how cluster‐scene connectivity influences local genomic instability. As the pattern transitioned from single‐pattern to multiple‐pattern, the nodes were increasingly enriched in promoter regions (Figure 4C), and the proportion of these nodes located within 0 to 1 kb upstream and downstream of transcription start sites gradually increased (Figure S7F, Supporting Information). Given that promoters are known to be one of the genomic fragile sites,^[^ ^39^ ^]^ this finding supports the inherent DSB susceptibility of highly connected hub nodes.

To gain deeper insights into the cluster‐scene mechanism between hub nodes and central DSB sites they connected, we conducted a detailed analysis of the genomic distances between these elements. Our results revealed that the average linear genomic distances between hub nodes and DSB sites they connected are significantly longer compared to other patterns (*p* < 0.0001, Wilcox test, Figure 4E,D left). This observation led us to hypothesize that the cluster‐scene phenomenon may be more closely related to the spatial positioning of these nodes within the 3D chromatin architecture, rather than being solely determined by their linear genomic distance.

To investigate this hypothesis, we aimed to accurately reflect the spatial positions of chromatin segments, including hub nodes and their connected DSB sites, in 3D space. We evaluated high‐resolution algorithms against three benchmarks: chromatin image‐based tracking data, original Hi‐C data, and simulated data (Figure 4F). Through comprehensive comparisons (Experimental Section), we determined that FLAMINGO^[^ ^40^ ^]^ was the most suitable method for reconstructing the spatial positions of genomic segments, as it demonstrated superior performance across various evaluation scenarios (Figure 4G–I).

3D Euclidean distance analysis of FLAMINGO‐reconstructed chromatin structures revealed spatial clustering patterns across interaction types. Hub nodes showed significantly shorter average Euclidean distances to connected DSB sites compared to single‐pattern (*p* < 0.0001, Wilcox test) and double‐pattern nodes (*p* < 0.0001, Wilcox test, Figure 4J and right panel of Figure 4D). This spatial preference was conserved in the K562 cell line (Figure S8G, Supporting Information), confirming our hypothesis that 3D proximity rather than linear genomic positioning as the primary determinant of cluster‐scene. Mechanistically, Loop facilitates closer interactions within specific regions, thereby reducing the spatial distance between anchored genomic elements.^[^ ^40^, ^41^ ^]^ Notably, in both NHEK and K562 cell lines, we observed that hub nodes were most frequently located at loop anchors (Figure S9F,G, Supporting Information), indicating that regions containing hub nodes are more prone to form chromatin loops, thereby increasing local chromatin interaction density, promoting spatial clustering of genomic segments, and ultimately facilitating DSB formation in these localized domains, which further help elucidates the cluster‐scene.

### 2.4 TCI Reveals Links Between Chromatin Architecture and Genomic Stability

**Figure 5:**
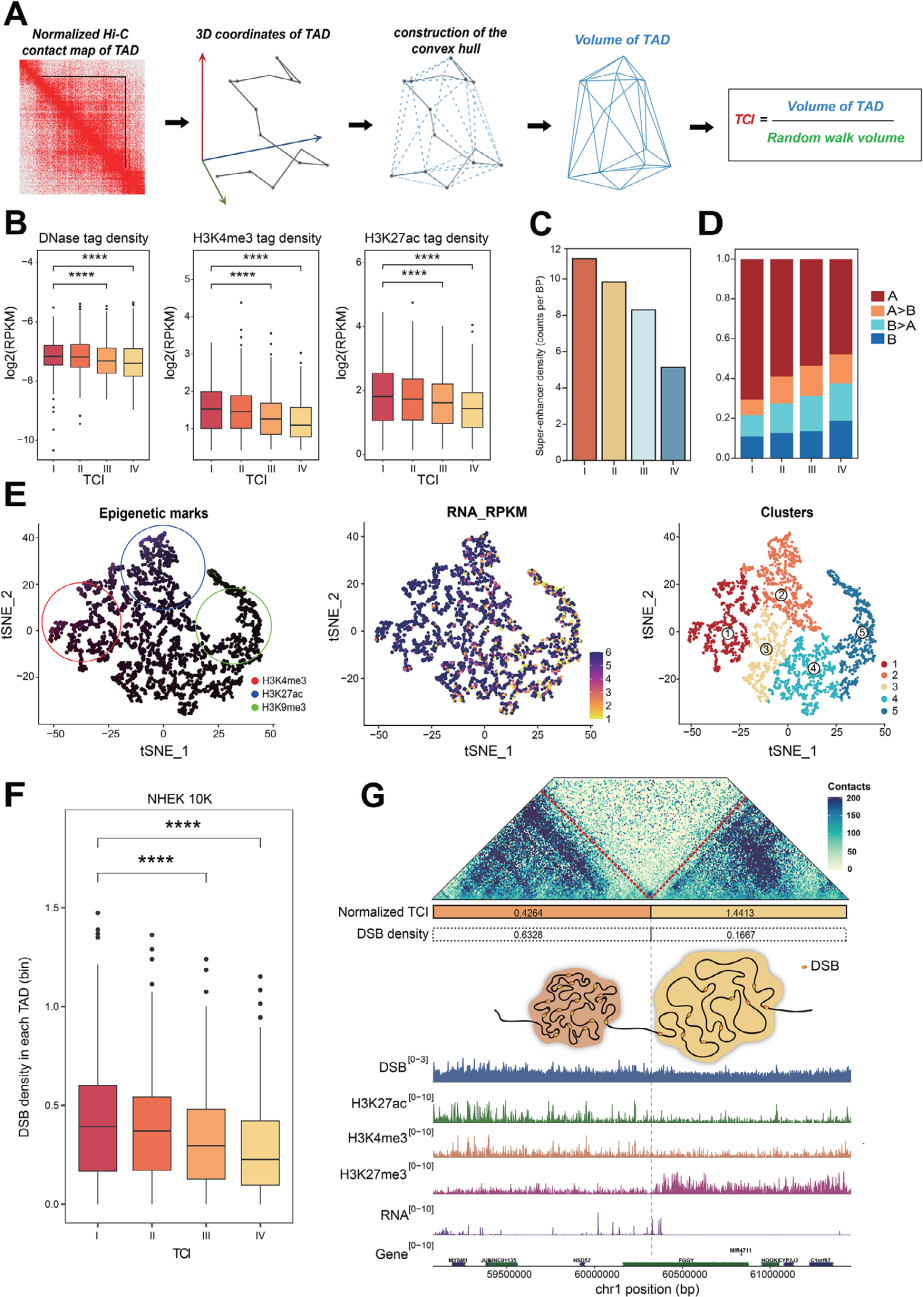
Calculation and properties of TCI. A) Schematic diagram of TCI calculation. B)Epigenetic signature for the four groups of TADs (I‐IV, divided by quantile‐normalized TCI fromlow to high). ^**^ *p* < 0.01, ^***^ *p* < 0.001, ^****^ *p* < 0.0001, Wilcox test. C) The density ofSuper‐enhancer in each group of TCI. D) Fraction of TADs with different compartmentcomposition in different groups of TCI, A > B denote the fraction of TADs with mixedcompartment (compartment A > 50% of TAD region), B>A (compartment B > 50% of TADregion). E) t‐SNE clustering plot. Left panel: Intensity in red, blue, and green corresponds tothe density of H3K4me3 tags, H3K27ac tag density, and H3K9me3 tag density within eachTAD, respectively. Middle panel: RNA‐seq tag density within each TAD. Right panel: TADclusters. F) Comparison of DSB density for four groups of TADs (I‐IV, divided byquantile‐normalized TCI from low to high) ^****^ *p* < 0.0001, Wilcox test. G) Top: An exampleHi‐C heatmap of a region (chr1:59080000‐61460000) in NHEK. Middle: An illustration of TADswith different TCI. Bottom: DSB, H3K27ac, H3K4me3, and H3K27me3 ChIP‐seq data on thespecified genomic region.

Based on the above analysis, we speculate that hub nodes not only participate in the higher‐order chromatin structure to promote DNA folding but may also influence the compactness of the TADs in which they reside. Here, we developed computational metrics to characterize the density of chromatin spatial interactions within TADs, termed the TAD Compactness Index (TCI) calculated as the ratio of observed TAD volume (**Figure** **5**A) to theoretical random‐walk volume for equivalent genomic lengths (Figure S10A, Supporting Information) (Experimental Section). A smaller TCI indicates a more compact TAD with a higher density of chromatin interactions. By normalizing the effect of TAD length on compactness, the TCI enables standardized comparisons of interaction densities across different TADs. Multi‐scale validation confirmed TCI sensitivity to chromatin organizational states (Experimental Section), establishing it as a length‐normalized metric for cross‐TAD compaction comparisons.

As basic regulatory units of genomic damage,^[^ ^42^ ^]^ TADs were analyzed for their regulatory and functional features using the TAD Compactness Index (TCI). We evaluated TCI at a 10 kb resolution and classified all TADs into four categories based on quantile‐normalized TCI values using thresholds (‐1, 0, 1) (Experimental Section). The TCI increases from the first to the fourth category, with the first category representing the smallest TCI values—and therefore the highest chromatin interaction intensity. When comparing these four TCI categories from low to high, we observed that the DNase signal intensity and active histone modification signal intensity, such as H3K27ac and H3K4me3, decreased with the increase of TCI (Figure 5B; Figure S10E, Supporting Information). Moreover, super‐enhancers associated with high transcript levels were significantly enriched in TADs belonging to the lowest TCI category (Figure 5C). Additionally, regions with smaller TCI exhibited a greater association with A compartments, indicating that the smallest TCI is linked with increased transcriptional activity and a bias toward the activation of compartment A (Figure 5D). These findings demonstrate that TCI effectively captures significant differences in genomic, epigenomic, and transcriptional activities across different TADs.

To further illustrate that TCI can be used to characterize the gene expression and epigenetic state of TADs, we employed t‐SNE for dimensionality reduction based on the epigenetic features of TADs (Figure 5E). We classified TADs into five categories using K‐means clustering based on H3K4me3, H3K9me3, and H3K27ac modifications, visualizing them in two dimensions and assigning different colors to represent various epigenetic marks. The color intensity corresponds to the level of gene expression, with variations indicating the diversity of the epigenetic states of TADs and correlating with gene expression levels (Figure 5E). TCI demonstrates distinct distributions across these five clusters (Figure S11B, Supporting Information), and the TCI‐associated grouping on the t‐SNE map also reflects differences in gene expression and epigenetic states (Figure 5E). These results support the functional characteristics of TAD as a regulatory unit and further validate the capability of TCI to characterize different epigenetic states.

The variation in multi‐dimensional properties within TADs suggests different susceptibilities to DSBs.^[^ ^23^ ^]^ To explore the potential link between chromatin spatial interaction density and DSB formation, we conducted an association analysis between the TCI and DSB sites. Given the variability in TAD lengths, we calculated the number of DSBs in each TAD and normalized this by the length of the TAD, defining it as DSB density to characterize the probability of DSB occurrence per unit length of TAD. Our results revealed a significant negative correlation between TCI and DSB density in both NHEK and K562 cell lines (Figure 5F; Figure S10F, Supporting Information), indicating that lower TCI values are associated with increased DSB susceptibility. This association appears to be independent of cell type, suggesting that it may reflect a common regulatory principle across cellular contexts. To validate the robustness of this finding, we controlled for TAD length as a potential confounding variable. Even after accounting for TAD length, TCI remained negatively correlated with DSB density (Figure S11C, Supporting Information) (Experimental Section). These findings support a relationship between TCI and DSB incidence, highlighting the critical role of TAD 3D spatial conformation in maintaining genome integrity. Our genome browser diagrams further illustrate differences in DSB frequency and epigenetic modifications across regions with varying TCI values, underscoring the heterogeneity in genomic stability and regulatory landscapes associated with distinct levels of chromatin compaction (Figure 5G).

These results reinforce the functional role of TADs as regulatory units and further validate the ability of TCI to capture distinct epigenetic states, highlighting its importance in understanding how the cluster‐scene influences genomic stability and function.

### 2.5 Chromatin Compaction and Boundary Insulation Contribute to Cluster‐Scene Formation in TADs

**Figure 6:**
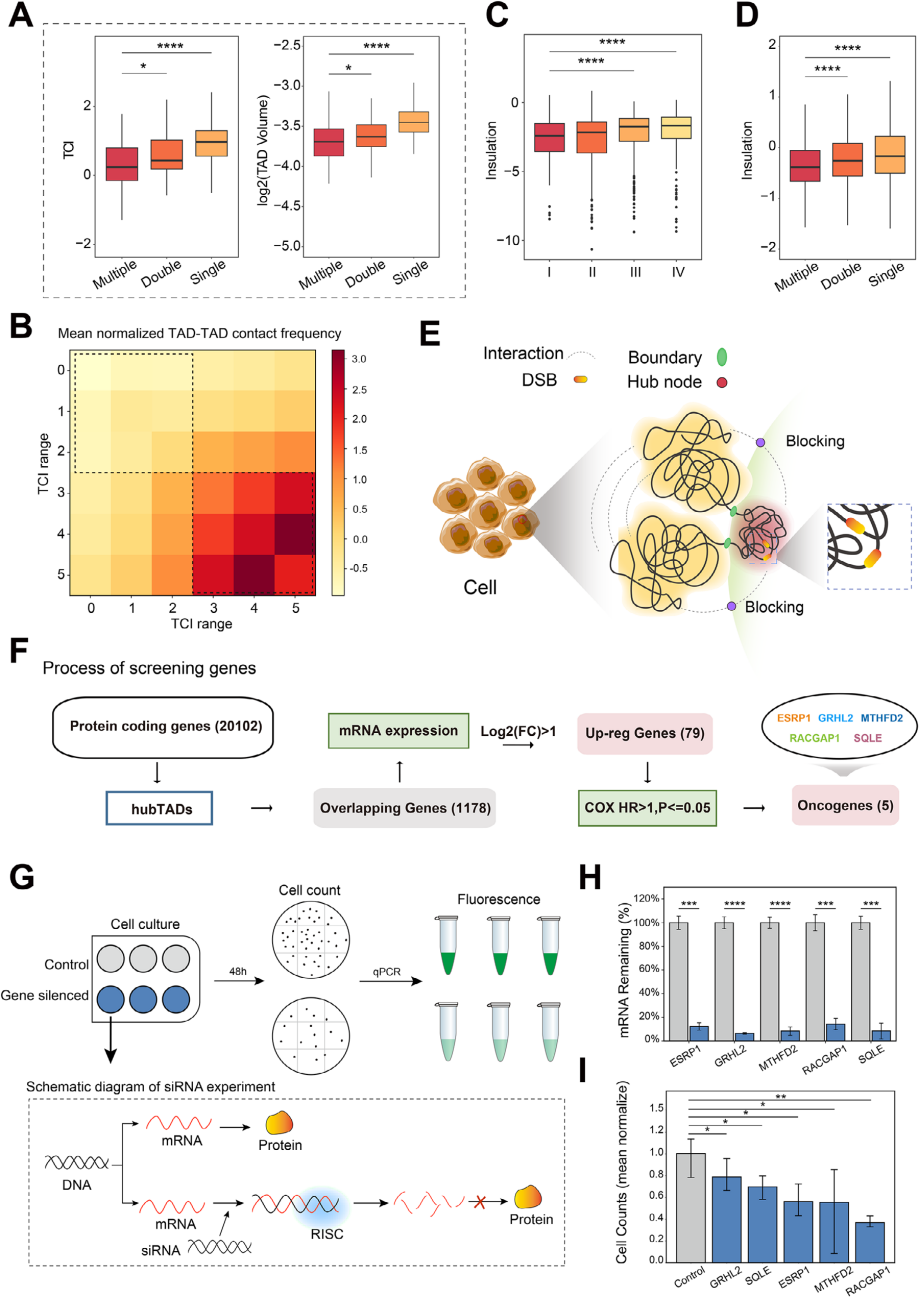
Spatial aggregation of chromatin and DSB sensitivity. A) Boxplots showingdifferent TCI among TADs under different patterns. ^*^ *p* < 0.05, ^**^ *p* < 0.01, ^***^ *p* < 0.001, ^****^ *p* < 0.0001, Wilcox test. B) Heatmap showing mean normalized TAD‐TAD contact frequency fromvarious TCI range. C) Comparison of insulation for four groups of TAD boundary (I‐IV, dividedby quantile‐normalized TCI from low to high) ^****^ *p* < 0.0001, Wilcox test. D) Differences ininsulation among TAD boundaries under different patterns. ^****^ *p* < 0.0001, Wilcox test. E)Schematic illustration of ‘Spatial Isolation–Damage Sequestration’. Red tangle: hub‐TADs(more compact, low TCI, DSB‐prone); Yellow tangle: other TADs (more relaxed, high TCI, DSB‐resistant); Green boundary: boundary insulator; Dashed box enlargement: spatialproximity between hub nodes and DSBs. F) Schematic flow chart of the screening processassociated within hubTADs, to obtain oncogenes genes. G) Schematic diagram of thesiRNA‐Mediated knockdown process for the five risk genes. H) qPCR confirmed reduction intarget mRNA levels for genes (*p* < 0.05, two‐tailed *t*‐test).I) Proliferation assays after siRNA perturbation (*p* < 0.05, two‐tailed *t*‐test).

Comparative analysis of chromatin folding modes revealed distinct TCI profiles from single‐pattern to multiple‐pattern TADs. Hub nodes enriched domains (hubTADs, n = 146; Experimental Section) demonstrated significantly lower TCI scores and the smallest volumes (**Figure** **6**A; Figure S10G, Supporting Information), supporting the role of hub nodes in promoting chromatin compaction. In addition, hubTADs displayed stronger internal interactions (Figure S11D, Supporting Information) and a higher proportion of loop anchors (Figure S9F,G, Supporting Information). The enrichment of loop anchors and enhanced intra‐TAD interactions suggest a more compact and highly interactive chromatin environment, highlighting the critical role of chromatin architecture in shaping genomic stability.

Boundary strength was hypothesized to modulate chromatin confinement effects. Quantitative analysis of normalized inter‐TAD contact frequencies across TCI quartiles showed weaker inter‐TAD contact frequencies in low‐TCI domains versus high‐TCI counterparts (Figure 6B). Class‐specific comparisons revealed that multi‐TAD clusters exhibited lower interaction strength than other TAD categories (Figure S11E, Supporting Information). Suggesting that differences in inter‐TAD interaction strength may be influenced by variations in boundary insulation. Stronger boundary insulation appears to help isolate damaged TADs from surrounding regions, thereby confining DSBs within specific chromatin domains and contributing to the formation of spatial cluster‐scene. To validate this hypothesis, we examined the boundary insulation of TADs across different TCI ranges. The results were consistent with our conjecture: TAD with lower TCI values exhibited stronger boundary insulation (Figure 6C; Figure S11F, Supporting Information). Notably, hubTADs showed both higher DSB susceptibility (Figure S11G, Supporting Information) and the strongest boundary insulation (Figure 6D). These findings not only confirm our hypothesis but also highlight the crucial role of boundary strength variations in regulating the spatial distribution of DSBs.

We propose that changes in TCI and boundary insulation metrics reflect structural adaptation to genomic stress. In U2OS cells, DSB induction triggered significant TAD compaction (Figure S12A,B, Supporting Information) and increased boundary insulation scores (Figure S12C Supporting Information) (Experimental Section). Validation in NHEK showed DSB‐containing TADs maintained lower TCI values and stronger insulation compared to length‐matched intact domains (Figure S12F,G, Supporting Information) (Experimental Section). Tumor progression modeling (MCF10A to MCF7) (Experimental Section) revealed malignant transformation‐associated chromatin restructuring, with DSB TADs exhibiting TCI reduction and insulation enhancement relative to non‐damaged counterparts (Figure S12D,E, Supporting Information). These orthogonal cellular models demonstrate that TCI and boundary insulation jointly quantify domain‐level structural responses to DNA damage, establishing their utility as spatial biomarkers for monitoring genome stability.

Here, we propose a “spatial isolation–damage containment” hypothesis to illustrate how the genome responds to DNA damage (Figure 6E). Within hubTADs, this model highlights the critical role of cluster‐scene between hub nodes and DSBs, which occurs predominantly at the spatial level. These hub nodes and DSBs are far apart in linear distance but close in spatial distance, likely due to high‐frequency contacts between genomic segments. Between TADs, hubTADs are characterized by smaller volumes, low TCI (corresponding higher interaction densities), and stronger boundary insulation. The boundary reinforcement reduces the frequency of inter‐TAD interactions, isolating the damaged TADs from their neighbors. Such isolation not only confines DSBs within specific chromatin domains but also contributes to the occurrence of cluster‐scene. This coupling of structure and function facilitates the formation of a microenvironment within hubTADs that is conducive to the recruitment of DNA damage repair factors (Figure S12H, Supporting Information), thereby promoting the DNA repair process.

### 2.6 Investigation of BRCA‐Related Genes in hubTADs

We propose that analyzing variations in gene enrichment within hubTADs can uncover functionally relevant molecular mechanisms. Toward this end, we designed a computational framework that combines differential expression analysis of BRCA tumors (from the TCGA cohort) with spatial chromatin architecture profiling to identify regulatory genes within these topological regions. (Figure 6F) (Experimental Section). This process led to the identification of 79 genes in DSB hubTADs with significantly up‐regulated expression levels. Univariate COX regression analysis identified 5 genes with hazard ratios (HR) > 1, considered to be risk genes (Figure S13A, Supporting Information).

The expression levels of genes *ESRP1, GRHL2, MTHFD2, RACGAP1*, and *SQLE*, considered risk factors, were significantly up‐regulated in tumor tissues compared with normal tissues (Figure S13B, Supporting Information) and were statistically significantly associated with a poor prognosis (Figure S13C, Supporting Information). It has been reported that overexpression of RACGAP1 plays a crucial role in tumor metastasis^[^ ^43^, ^44^ ^]^ and drives metastasis in breast cancer.^[^ ^45^, ^46^ ^]^

Functional validation of the five risk genes (*ESRP1, GRHL2, MTHFD2, RACGAP1, SQLE*) was performed through siRNA‐mediated knockdown in MCF7 cells (Figure 6G) (Experimental Section). qPCR confirmed ≥70% reduction in target mRNA levels for all genes (Figure 6H; Table S7A, Supporting Information). Proliferation assays revealed that silencing *ESRP1, MTHFD2, RACGAP1, GRHL2*, and *SQLE* significantly inhibited proliferation (20–62% reduction vs controls, p < 0.05, two‐tailed t‐test) (Figure 6I; Table S7B, Supporting Information), These results establish the contribution of these genes to breast cancer cell growth and highlight their potential as functional candidates in breast cancer development.

We further investigated the five risk genes from the perspective of 3D chromatin architecture to reveal their spatial characteristics and potential regulatory mechanisms. These loci exhibited hallmark hubTAD features, including dense clustering of active chromatin marks (H3K27ac, H3K4me3) and elevated DSB susceptibility (Figure S14A,B, Supporting Information). Chromatin interaction analysis revealed connectivity between candidate gene loci and architectural hubs (Experimental Section), with interaction frequencies higher than non‐hub node pairs (Figure S14C, Supporting Information). Spatial proximity quantification via Hi‐C derived Euclidean distances confirmed shorter physical associations between candidate loci and hub nodes (Figure S14D, Supporting Information), establishing a “high‐frequency, short‐distance” interaction paradigm. This 3D configuration supports loop‐like mediated transcriptional regulation and offers structural evidence for the coordinated activation of genes within hubTADs. It also establishes a structural basis for the cluster‐scene phenomenon observed in these regions.

Therefore, we believe that analyzing hubTADs regions can help elucidate the causes of transcriptional abnormalities suggesting that these regions play a potential role in linking genomic instability. Overall, the screening of these cancer genes using our model could provide new insights into the mechanisms underlying genome stability in the context of complex 3D chromatin organization.

## 3 Discussion

In summary, we present ChromInSight, a computational framework for systematic characterization of DSB loci. This platform systematically analyzes DSB hotspots through an integrative analytical framework that incorporates sequence features, histone modification patterns, and 3D chromatin architecture. The framework integrates standardized DSB datasets with Hi‐DSB, a graph contrastive learning architecture that resolves genome‐wide DSB prediction while addressing intrinsic genomic imbalance and using AUROC and AUPRC as performance metrics, thereby enhancing quantifiable comparisons among models. Benchmarking against existing methods across multiple cell lines and sequencing resolutions, Hi‐DSB achieves competitive performance, demonstrating enhanced precision in genome instability profiling.

Additionally, we used advanced graph network explainability techniques – GNNExplainer, to identify the most critical nodes for predicting DSBs and classified them into three categories based on the number of DSBs they connect to: single‐pattern, double‐pattern, and multiple‐pattern. From the perspective of chromatin 3D structure, we evaluated existing methods for reconstructing the spatial conformation of chromatin fragments, aiming to more precisely reflect the spatial distances between hub nodes and DSB sites. By calculating the Euclidean distance between hub nodes and DSB sites, we revealed that the cluster scene between hub nodes and DSB sites is primarily dependent on chromatin spatial conformation rather than proximity of linear genomic distance.

We validated the cluster‐scene phenomenon across chromatin loops and TAD hierarchies. Chromatin looping elevates interaction density, driving specific genomic region aggregation. Hub nodes demonstrated preferential localization at loop anchors, confirming their looping competence. This structural propensity increased local interaction density, which in turn enhances spatial clustering and DSB occurrence. TAD‐level analysis employing TCI revealed mechanical stratification. DSB‐enriched hubTADs displayed enhanced compaction, reduced spatial volume, and reinforced boundary insulation. These architectural features confined DSB propagation while promoting repair factor recruitment, establishing TADs as functional units for damage containment and resolution.

Hi‐DSB integrates chromatin 3D architecture and epigenomic features to predict genome‐wide DSB loci with high precision. While cross‐cell line validation revealed reduced predictive accuracy, this limitation aligns with intrinsic biological divergences in chromatin topology, epigenetic landscapes, and cell‐type‐dependent DSB patterning. Notably, Hi‐DSB maintained superior performance compared to existing DSB predictors in cross‐cell evaluations, demonstrating generalizability across cellular contexts. Future development will focus on enhancing cross‐cell generalizability through graph contrastive learning frameworks trained on the integration of pan‐cell‐line epigenomic datasets. For cell types with unique DSB patterns, we will apply transfer learning and fine‐tuning—using a multi‐cell‐type model as a foundation and refining it with limited target‐cell‐specific data. In parallel, risk gene prioritization will strategically integrate TCI metrics with transcriptional profiles. TCI quantifies TAD‐level structural heterogeneity while correlating with DSB susceptibility, establishing its utility in mapping structural determinants of genomic vulnerability. Making it a biologically meaningful metric for studying 3D genome regulation cancer.

Advances in sequencing technologies now enable chromatin spatial profiling at improved resolution, synergizing with multiomics integration to advance understanding of 3D genome organization. Current studies predominantly rely on Hi‐C data at 10–40 kb resolution, which inadequately resolves fine‐scale structural dynamics within TADs and their functional coupling to DSB formation. Furthermore, the scarcity of longitudinal Hi‐C datasets from pre‐ and post‐treatment patient cohorts hinders the analysis of dynamic TAD reorganization during disease progression or adaptation.

Integrating high‐resolution Hi‐C data (like base‐pair resolution) with expanded longitudinal sampling would enhance precision in mapping chromatin structural determinants of DSB susceptibility. Concurrently, advances in deep learning architectures, particularly expressive neural networks designed for spatial omics analysis, are expected to further refine predictive accuracy by modeling hierarchical chromatin interactions. Self‐supervised graph learning frameworks demonstrate the capacity to derive transferable chromatin representations through systematic analysis of unannotated genomic datasets. Multimodal integration architectures, particularly those combining transcriptomic signatures with protein interaction landscapes, enable systematic decoding of multilayered regulatory circuits governing DSB biogenesis. Interpretable graph attention mechanisms further permit the identification of causal relationships between regulatory hubs and DSB hotspots, directly linking topological chromatin features to genome stabilit mechanisms.

In summary, combining high‐resolution 3D genome data with advanced deep learning techniques not only enhances the accuracy and biological interpretability of DSB prediction but also opens new avenues for investigating the mechanisms of genomic instability within complex 3D genome architectures.

## 4 Experimental Section

### Data Source

The table below shows all DSB data sources used. All GEO numbers are displayed in accession numbers.(**Table** **1**)

**Table 1:** DSB datasets.

Cell line	Process	Source	Article
NHEK	No treatment	DSBCapture	Nat Methods. 2016 PMID: 27525976
NHEK	No treatment	BLESS	Nat Methods. 2016 PMID: 27525976
K562	No treatment	BLISS	Genome Biol. 2019 PMID: 30 736 820
K562	Treatment	BLISS	Genome Biol. 2019 PMID: 30736820
MCF7	No treatment	BLISS	Genome Biol. 2019 PMID: 30736820
Nalm6	No treatment	END‐seq	Cell. 2017 PMID: 28735753
Nalm6	Treatment	END‐seq	Cell. 2017 PMID: 28735753

### Sequence Analysis

DSBCapture libraries: A detailed DSBCapture sequence analysis were published.^[^ ^6^ ^]^ In short, adaptor sequences were removed by trim_galore (http://www.bioinformatics.babraham.ac.uk/projects/trim_galore/). Reads were aligned to the human reference genome (hg19) using BWA mem (http://sourceforge.net/projects/bio‐bwa/files/). Use samtools (http://samtools.sourceforge.net) to clean reads (mapQ <10) and picard tools to remove duplicates.

BLESS libraries: A detailed BLESS sequence analysis were published.^[^ ^6^ ^]^ Briefly, trim the BLESS linker sequences (TCGAGGTAGTA and TCGAGACGACG). Only linkers both containing read1 and read2 were reserved, and trimmed reads then undergo the same processing pipeline as DSBCapture libraries.

END‐seq libraries: Fastq files containing sequencing reads were pre‐processed to remove the Illumina adapters and trim low‐quality tails using trim_galore (http://www.bioinformatics.babraham.ac.uk/projects/trim_galore/). Tags were aligned to the human reference genome (hg19) using the BWA mem (http://sourceforge.net/projects/bio‐bwa/files/) and the alignment output sam files were converted into bam files using samtools.

BLISS libraries: The same workflow was operated for pre‐processing BLISS sequencing data.^[^ ^8^ ^]^ In brief, R1 reads starting with UMI and barcode, ‐1 mismatch allowed in UMI and barcode. Then extract the genomic sequence from R1 reads with UMI and barcode. After the removal of the prefix, the extracted genomic sequence was aligned to the reference genome (hg19) using the BWA mem aligner.

### Peak Calling

Peaks for DSBCapture and BLESS can be easily taken from the reference by Stefanie V. Lensing et al^[^ ^6^ ^]^ under GEO accession GSE78172. Since the peaks of BLISS and END‐seq were not available from the GEO database, peak‐calling needed to be operated. Therefore, the raw data of BLISS and END‐seq were subjected to quality control. Since DSBCapture and BLESS peaks were compared to the hg19 genome version. To facilitate comparison with DSBCapture and BLESS peaks, the effective reads of END‐seq were screened and compared to the human hg19 genome version. Then peak‐calling was performed using the softwares and parameters required in the respective articles (Table S1, Supporting Information). For BLISS, it was worth noting that after align to the reference genome (hg19), the same workflow for pre‐processing sequencing data as described in Yan et al was continued to be used,^[^ ^8^ ^]^ including filter mapped R1 and filter UMIs, and finally generate BED files. Since BLISS can be processed by using custom scripts, the script of bliss_align.sh used in Tracy Ballinger et al^[^ ^32^ ^]^ was executed to process BLISS data. By executing bliss_align.sh, the final BED file containing unique UMIs could be obtained.

### Bam to BigWig

BamCoverage in deeptools (https://deeptools.readthedocs.io/en/develop/) was used to generate the bigwig file, with the parameter of –normalizeUsing RPKM.

### Plot Heatmaps

Heatmaps were generated by using deep tools (such as multiBamSummary, computeMatrix, plotHeatmap, plotCorrelation and plotProfile).

### ChIP‐seq Visualization

Deeptools (https://deeptools.readthedocs.io/en/develop/) was used for visualization and signal enrichment analysis of ChIP‐seq data. Specifically, the computeMatrix tool records the DSB enrichment peaks based on the coordinates and derives the signal enrichment heatmap from the Bigwig file, which was then plotted using the plotHeatmap and plotProfile tools.

### Hi‐C Data Processing

Hi‐C data for three human cell lines (K562/NHEK/MCF7) were used. AllValidPairs files could be used to build raw Hi‐C contact matrix files (in sparse matrix format) at three resolutions: 10, 25, and 50 kb resolution. KR (Knight–Ruiz) and MCFS (Median Contact Frequency Scaling) methods were used to implicitly correct the contact matrix. Then HiC‐Pro^[^ ^47^ ^]^ was used to convert sparse matrix to dense matrix.

### Hi‐C Normalized with KR and MCFS

Juicer_tools ‐dump ‐observe ‐KR was first used to perform Knight‐Ruiz (KR) normalization on the Hi‐C matrix, which balances rows and columns and corrects for variations in coverage between bins. Subsequently, MCFS normalization was applied using gcMapExplorer normMCFS (https://gcmapexplorer.readthedocs.io/en/latest/commands/normMCFS.html) to remove contacts based on the average contact probability as a function of genomic distance, thereby improving the accuracy of the Hi‐C data for downstream analysis.

### Detection of Contact Domains

TADs were calculated using insulation score with a bin size of 10, 25, and 50 kb. The mean value of the interactions between each bin was computed. Then with Hi‐C interactions, tTAD boundaries were obtained using matrix2insulation.pl. Insulation vectors were detected using the following options (‐is 800 000 ‐ids 200 000 ‐im mean ‐bmoe 0 ‐nt 0.1 ‐v). TAD regions were further calculated between each of the adjacent boundaries.

### Building Hi‐DSB

A representation learning algorithm based on graph contrastive learning is proposed, which effectively captures graph structure and node feature information through a self‐supervised learning mechanism to enhance the predictive performance of downstream tasks. The model integrates Graph Attention Networks (GAT) and contrastive learning strategies, employing multi‐level embedding generation and optimization to achieve fine‐grained node representation learning. The overall workflow consists of four main components: input, embedding generation, contrastive learning, and output, with each module working in concert to achieve efficient graph representation learning.

### Model Input–Construction of the Original Graph

The graph was constructed using 3D chromatin contact information (Hi‐C), consistent with the method used for constructing DSB‐GNN graphs. The interaction information from Hi‐C was used as edge weights, and genomic features such as CTCF, DNase, H3K27ac, and H3K4me3 were used as node features. Specifically, each chromosome was modeled as an undirected weighted graph. The genome was divided into equal bins at a 10 kb resolution, with each bin corresponding to a genomic region being treated as a node, and the chromatin interactions between bins as edges. Node features were defined as the number or signal intensity of histone modification peaks within a specific genomic bin. Each graph was denoted as G∈(V, E), where V was the set of nodes vi in the graph, and E was the set of edges ei. Each node vi has an initial feature vector hi, which constitutes the node feature matrix $H\;\in \;R^{\left\{|V|\times d\right\}}$, where d represents the dimensionality of the features.

### Nodes Shuffle

To enhance the robustness and generalization capabilities of the model, node perturbation was first applied to the original graph G, generating a corresponding corrupted graph *G*′= (*V*,*E*,*H*′). Node perturbation was achieved by randomly shuffling the node features, which simulates the diversity and noise in graph structures, thereby encouraging the model to learn more stable and robust node representations. This reordering of nodes can be represented using a permutation matrix P. P was a binary matrix of size |V| x |V|, where each row and each column contains exactly one element equal to 1, with all other elements being 0. It satisfies the following properties:

(1) $$P\;P^{T}=P^{T}\;P=I$$

where *I* denote the identity matrix. This property indicates that *P* is an orthogonal matrix, and its inverse was equal to its transpose. The process of node perturbation can be mathematically described as applying the permutation matrix P to the original node feature matrix H: *H*′ =  *PH* Here, P is generated randomly while ensuring it meets the criteria of a permutation matrix, and *H*′represents the feature matrix after node perturbation.

### Node Embedding Generation via GATs

The embedding generation module utilizes GATs to process both the original graph G and the corrupted graph *G*′, producing corresponding node embeddings H and *H*′, respectively.

### Graph Attention Networks

GATs use a self‐attention mechanism to assign different attention weights to each node, thereby dynamically aggregating information from neighboring nodes. The attention mechanism for a node and its neighbors in GAT can be expressed as:

(2) $$e_{ij}=LeakyReLU\left(a^{T}\left[Wh_{i}|Wh_{j}\right]\right)$$

Where:

*h_i_* and *h_j_* are the initial feature vectors of nodes i and j, respectively.
*W* is a linear transformation matrix.
*a* is the weight vector in the attention mechanism.
| denotes the concatenation operation.
Next, the attention coefficients are normalized using the Softmax function:
(3) $$a_{ij}=\frac{\exp\left(e_{ij}\right)}{\sum \left(k\in N\left(i\right)\exp\left(e_{ik}\right)\right)}$$

Finally, the new embedding representation of node *i* is given by:

(4) $$h_{i^{\prime }}=\sigma \left(\sum_{j\in N\left(i\right)}a_{ij}w^{h_{j}}\right)$$

Where σ is a non‐linear activation function.

### Contrastive Learning

Contrastive learning was introduced to optimize the discriminative power of node embeddings by maximizing the similarity between the same nodes across different views while minimizing the similarity between different nodes. The specific process includes the following steps:

### Readout Operation

The readout operation was used to generate a central graph representation *z_c_* from the node embeddings. An alignment mechanism was employed between the embedding of the central node and the central graph representation to ensure that the central graph representation was close to the central node's representation, while the corresponding corrupted graph representation should be distant from the central node's representation. The central graph representation was generated by aggregating the embeddings of the neighboring nodes. The central graph representation *z_c_* was computed as follows:

(5)

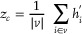

Where:

*v* is the set of neighboring nodes.
h_*i*_′ is the embedding of node *i* in the perturbed graph.
*h_c_* is the embedding of the central node.
*z_c_* is the central graph representation.

### Contrastive Loss Function

The contrastive learning part adopts the InfoNCE loss function, which aims to maximize the similarity between positive sample pairs (the central graph representation and the central node representation) while minimizing the similarity between negative sample pairs. The specific loss function was defined as follows. The dot product or cosine similarity was used as the similarity measure:

(6) $$sim\left(a,b\right)=\frac{a^{T}b}{\left|a\right|\left|b\right|}$$

InfoNCE loss function:

(7)

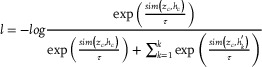

Where *z_c_* is the embedding representation of the central graph obtained through the readout operation, *h_c_* is the embedding representation of the central node, $h_{k}^{\prime }$ represents the embeddings of the negative sample nodes. τ is a constant that adjusts the smoothness of the similarity distribution.

This loss function achieves the aggregation of similar samples and the separation of different samples by maximizing the difference between *sim*(*z_c_*,*h_c_*) and $sim(z_{c},h_{k}^{\prime })$. Its goal can be decomposed into maximizing the similarity of positive sample pairs and minimizing the similarity of negative sample pairs.

### DSB prediction

After the optimization through contrastive learning, the refined node embeddings H* were obtained. These embeddings not only preserve the local features of the nodes but also integrate information from the central graph structure. The final step was to use these embeddings for DSB prediction. Consistent with the method used for DSB‐GNN the classifier outputs a probability or a binary decision indicating the likelihood of a DSB occurring at each node.

### Explainability Principles of GNNExplainer

GNNExplainer was a method designed to provide interpretability for various Graph Neural Network (GNN) models. It works by maximizing the mutual information between the predicted label and the subgraph structure as well as the subset of node features. This approach helps identify the most important subgraphs and feature subsets that contribute to the prediction, making it particularly effective in explaining complex biological models.

Specifically, for a node *v* the model's prediction of its label is given by

(8) $$\hat{y}=\emptyset \left(G\left(v\right),X\left(v\right)\right)$$

Where:

*G*(*v*) is the subgraph involving all nodes and edges relevant to *v* during the computation.

*X*(*v*) is the set of features associated with node *v*.

∅ represents the operations of the graph model.

GNNExplainer aims to identify the most important subgraph *G_s_* (*v*) ⊆ 𝐺(𝑣) and the most important feature subset *X_s_* (*v*) ⊆ 𝑋(𝑣) that were crucial for the prediction $\hat{y}$ The goal was to maximize the mutual information between the predicted label *Y* and the subgraph and feature subsets:

(9) $$max\;MI(Y,(G_{s}\left(v\right),X_{s}\left(\upsilon \right)=H\left(Y\right)\left|G=G_{s}\left(\upsilon \right)\right.,X=X_{s}\left(v\right)$$

Since 𝐻(𝑌) is a constant, the maximization problem was equivalent to minimizing the conditional entropy 𝐻(𝑌|𝐺 = *G_s_* (*v*), 𝑋 = *X_s_* (*v*)). GNNExplainer used a masking strategy to determine the subgraph *G_s_* (*v*) and feature subset *X_s_* (*v*). It learns masks for the edges and features, which were then used to compute the importance scores. Based on the learned masks, GNNExplainer assign importance scores to the edges and features.

### Model Ablation Experiments

To assess the contributions of genomic features and model components to Hi‐DSB's predictive performance for DSBs (Table S5, Supporting Information), an ablation study was conducted from three perspectives:

### Node Feature Analysis

Combinations of node features were considered and one feature at a time systematically removed to evaluate its impact on model performance. This approach allows us to identify which specific node features were most critical for accurate DSB prediction.

### Interaction Information Evaluation

Given that Hi‐DSB relies on Hi‐C interaction data to construct its graph, simply removing the Hi‐C network would render the model inoperable. To perform an effective ablation on Hi‐C data, the original Hi‐C input data were downsampled at various levels. By reducing the interaction information available to the model, this method helps us evaluate how changes in information retention affect Hi‐DSB's robustness and performance.

### Model Component Assessment

To quantify the contribution of each component within Hi‐DSB, several experiments were performed to compare model performance before and after removing or modifying key elements:

### Graph Contrastive Learning Component

Hi‐DSB was compared with a model based on a two‐layer GAT to test the impact of the graph contrastive learning framework.

### Graph Attention Mechanism

By comparing models with and without graph attention mechanisms, their role in capturing complex relationships between nodes was assessed.

### Skip Connections

The effect of skip connections on model performance was examined to evaluate their importance in facilitating information flow and feature integration.

### DSB Pattern Classification

The genome‐wide bins could be categorized into two classes: one class consists of bins with DSBs, labeled as 1 (referred to as central DSB sites); the other class comprises non‐DSB bins, labeled as 0 (referred to as central Non‐DSB sites). Focusing on the central DSB sites, for each central DSB site, GNNExplainer was employed to evaluate the importance scores of edges influencing that site. From these scores, the two nodes (key nodes) connected by the edge with the highest importance score were identified. These keys nodes were considered critical for determining the state of the current central DSB site. Notably, these key nodes not only play a pivotal role in determining the state of the current central DSB site but may also influence other central DSB sites. Based on the number of central DSB sites influenced by these key nodes, three distinct patterns were defined:

Single‐Pattern (single‐pattern): key nodes that were connected to a single central DSB site.

Double‐Pattern (double‐pattern): key nodes that were connected to two central DSB sites.

Multiple‐Pattern (multiple‐pattern): key nodes that were connected to three or more central DSB sites. These nodes were referred to as hub nodes, highlighting their significant role in the network structure.

It was important to note that these classification patterns were also applicable to the central Non‐DSB nodes (Figure S7A, Supporting Information).

Single‐Pattern (single‐pattern): key nodes that were connected to a central Non‐DSB node.

Double‐Pattern (double‐pattern): key nodes that were connected to two central Non‐DSB nodes.

Multiple‐Pattern (multiple‐pattern): key nodes that were connected to three or more central Non‐DSB nodes. These nodes were referred to as hub Non‐DSB nodes.

### Loop Anchors Defined and Detected

A classic model for loop formation was the loop extrusion model, which proposes that cohesin squeezes along the chromatin, creating progressively larger loop structures until it encounters two convergently oriented CTCF sites, where it stops. These two convergent CTCF sites with non‐palindromic sequences serve as loop anchors. The structures generated through loop extrusion can be detected using high‐throughput chromatin conformation capture techniques.Peakachu a random forest method for loop detection in genome‐wide contact maps was used, which were widely applied in detecting the chromatin loops in 56 Hi‐C datasets.

### Calculate the hubTADs

For TADs in the genome, They were classified based on the extent of their overlap with hub bins. Taking the calculation of hubTADs at 10Kb resolution as an example, to maximize the characteristics of hub nodes, a TAD was defined as one that must contain at least 10 or more hub nodes. The parameter bedops ‐e 10 000 was used to determine the hubTADs. The other TADs were calculated in the same manner.

### Install and Run 3D Reconstructed Spatial Model

FlAMINGO: The installation and run code link can be found in https://github.com/wangjr03/FLAMINGO.

EVR: The installation and run code link can be found in https://github.com/mbglab/EVR.

miniMDS: The installation and run code link can be found in https://github.com/seqcode/miniMDS

Pastis_NB: The installation and run code link can be found in https://github.com/hiclib/pastis

### Benchmarking of 3D Reconstructed Methods

A critical aspect of 3D genomics involves constructing the spatial structure of chromosomes by determining the spatial coordinates of each chromosomal DNA segment. Quantitative characterization of chromatin spatial structure was essential for understanding the coordinated regulation of the genome in space, facilitating transcription, and enabling epigenetic modifications.^[^ ^48^, ^49^ ^]^ To investigate the impact of 3D chromatin spatial structure on the occurrence of DSBs in cells, it was imperative to first evaluate various reconstruction methods to ensure they accurately reflect the spatial conformation of chromatin. Several algorithms have been developed to infer the spatial structure of chromosomes from ensemble Hi‐C data. These algorithms offer diverse perspectives on understanding chromatin spatial structure at different resolutions.^[^ ^40^, ^50^, ^51^ ^]^ Primarily utilizing the Hi‐C interaction matrix as input, these methods generate outputs in PDB or “xyz” formats, containing spatial coordinates along with bin structural information.^[^ ^40^, ^52^, ^53^ ^]^ However, some 3D reconstruction algorithms focus mainly on predicting genome structure at lower resolutions (≥100K) and specific genomic segments,^[^ ^52^, ^54^, ^55^ ^]^ whereas others can reconstruct chromatin 3D coordinates at a finer scale and higher resolution (10K‐50K).^[^ ^40^, ^56^, ^57^, ^58^ ^]^ Given that TADs represent finer structural units within the genome, more sophisticated reconstruction methods were required to accurately reflect their spatial organization. Thus, high‐resolution chromatin 3D structure reconstruction methods (10–50K), including FLAMINGO,^[^ ^40^ ^]^ miniMDS,^[^ ^57^ ^]^ Pastis‐NB,^[^ ^58^ ^]^ and EVR were compiled.^[^ ^56^ ^]^ The performance of each method was evaluated based on three criteria: (Chromatin image‐based tracking data, Original Hi‐C, Simulated data) (Figure 4F). Their accuracy and robustness were assessed by calculating the Root Mean Square Deviation (RMSD) and Coordinate Error (See Method).

### Performance on Chromatin Image‐Based Tracking Data

High‐resolution chromatin image‐based tracing data for a 2 MB genomic region on chromosome 21 from different cell lines (A549, HCT116, K562, IMR90)^[^ ^59^ ^]^ (XiaoWei Zhuang et al) were utilized as a gold standard. Single‐cell chromatin image‐based tracking data from these cell lines were processed, applying a threshold to exclude cells with more than 5% missing coordinates. Only cells with less than 5% missing data were retained, and linear interpolation was used to fill in the missing coordinates (*n*_*A*549_ =  497, *n*_*HCT*116_ =  420, *n*_*IMR*90_ =  694*, n*_*K*562_ =  112). To remove technical biases while preserving the core features of the original 3D genome architecture—thereby enabling structural comparisons and quantitative analyses across cells on a consistent scale, each single‐cell coordinate was centered and scaled using the F‐norm (“torch.nn.functional.normalize”). The results indicate that the average ensemble spatial‐distance matrices were consistent with the centering and F‐norm normalization process distance maps (Figure 4G and S7G, Supporting Information), suggesting that the normalization process preserves the chromatin contact patterns within individual cells while enabling meaningful structural comparisons across different cells. Subsequently, the performance of the reconstruction models was evaluated by calculating the RMSD and Coordinate Error between the reconstructed coordinates and the actual coordinates. This assessment demonstrated that, among the four cell lines tested, FLAMINGO consistently exhibited higher accuracy compared to the other three high‐resolution reconstruction methods (Figure 4I and Figure S7H, Supporting Information). The cell‐average spatial distance matrices generated by each reconstruction method revealed that FLAMINGO's output was closest to the super‐resolution chromatin image‐based tracing distance matrix (Figure 4H). Correlation statistics at each position in the cell‐average spatial distance matrices obtained by the four reconstruction methods, compared to the corresponding positions in the super‐resolution chromatin tracking distance matrix, further underscored the superior performance of FLAMINGO in reconstruction (Figure S8A, Supporting Information). TAD boundaries identified by the median spatial distance matrix inferred from the imaging data by FLAMINGO were highly similar to those determined by Hi‐C data, illustrating its accuracy and reliability in TAD boundary identification (Figure S8B, Supporting Information). In terms of single‐cell reconstructions, the illustrative figures highlight the superior performance of FLAMINGO (Figure S8C, D, Supporting Information). These findings indicate that FLAMINGO can effectively capture the natural spatial structure of individual chromatin fragments in a single cell.

### Performance On Original Hi‐C Contact

Hi‐C datasets from humans and yeast were utilized to demonstrate the robustness of these models in reconstructing performance across different species. Specifically, matrices generated by different reconstruction methods were compared with the actual Hi‐C interaction matrices to assess the performance of these models in reconstructing authentic Hi‐C interaction patterns. FLAMINGO exhibited patterns closely resembling the original Hi‐C interaction matrices (Figure S8E, F, Supporting Information). To further validate the general patterns of distance and interactions reconstructed by FLAMINGO, original Hi‐C data from the GM12878 cell line (GSE63525) at a 5 kb resolution across different genomic regions (chr21:30130000‐31080000, chr21:31375000‐32985000) were used. By calculating the chromatin spatial distances and interaction frequencies reconstructed by FLAMINGO, the results consistently demonstrated a significant negative correlation between chromatin contact frequency and genomic spatial distance (Figure S9A, B, Supporting Information). This finding aligns with previous research^[^ ^60^ ^]^ and supports the reliability of FLAMINGO for reconstructing 3D chromatin spatial structures.

### Performance On Simulated Data

The performance of these methods was evaluated using simulated data^[^ ^40^ ^]^ as input, allowing the comparison of the reconstructed 3D coordinates with benchmark coordinates through the calculation of RMSD and Coordinate Error. The results demonstrate that, compared to the other three methods, FLAMINGO maintains a higher level of accuracy, with its reconstructed results closely approximating the initial benchmark structure (Figure S9C, D, Supporting Information). Additionally, FLAMINGO exhibits optimal performance across varying numbers of genomic loci, further supporting that its accuracy was not affected by the number of genomic fragments among chromosomes^[^ ^40^ ^]^ (Figure S9E, Supporting Information).

### Calculate the Average Euclidean distance

For the chromatin segment with a number of loci represented by coordinates (xyz), assuming there were n loci, there will be a set of n^*^3 coordinates. First, this set of coordinates was centralized by finding the center point (*x*_0_, *y*_0_, *z*_0_). Then, the Euclidean distance for each locus (*x_i_*, *y_i_*, *z_i_*) to the center point (*x*_0_, *y*_0_, *z*_0_) was calculated. Finally, these distances and calculate the average was calculated.

(10) $$\mathrm{Average}\;\mathrm{Euclidean}\;\mathrm{distance}=\sum_{i=1}^{n}\frac{\sqrt{{\left(x_{i}-x_{0}\right)}^{2}+{\left(y_{i}-y_{0}\right)}^{2}+{\left(z_{i}-z_{0}\right)}^{2}}}{n}$$

### Performance of 3D Reconstructed Spatial Model

Different measures for simulating and chromatin image‐based tracking data were used to evaluate the performance of different 3D reconstruction algorithms. Since the original structure was known for simulating and chromatin image‐based tracking data, one measure was the Root Mean Square Deviation (RMSD). RMSD measures the similarity between two structures by calculating the pairwise coordinate distances of each loci between them.^[^ ^61^, ^62^ ^]^ RMSD is calculated as^[^ ^63^ ^]^:

(11) $$\mathrm{RMSD}\;=\sqrt{\frac{1}{N}\sum {∥R_{i}-P_{i}∥}_{2}^{2}}$$

Given a real N^*^3 3D coordinate (xyz) R = (r1, r2, …, rN) and a predicted structure with N^*^3 3D coordinates (xyz) P = (p1, p2, …, pN). Here, Ri and Pi represent the 3D coordinates (xyz) of the *i*th loci, where *i* = 1, 2, …, N. The smaller the RMSD value, the higher the similarity between these two structures, indicating better performance of the tested algorithm. Another metric was coordinate error, which evaluates the performance of these models by comparing the reconstructed structure with the true structure. The Coordinate Error was calculated as:

(12)

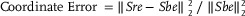

Where *Sre* represents the reconstructed 3D structure, and *Sbe* represents the benchmark 3D structure. Similar to RMSD, the smaller the value of the Coordinate Error, the closer the reconstructed structure was to the true structure, demonstrating better performance of the reconstruction method.

### TAD Volume Calculation

The contact matrix of each TAD was extracted from the interaction frequency KR and MCFS matrix of Hi‐C by the Insulation Score method^[^ ^64^ ^]^ as the input of the chromatin conformation reconstruction algorithm. Subsequently, the chromatin 3D spatial structure in each TAD was reconstructed by the FLAMINGO algorithm^[^ ^40^ ^]^ and then the corresponding 3D spatial coordinates of each genomic region were obtained. After that, in each TAD, the 3D structure of the TAD was constructed by the set of points composed of all 3D spatial coordinates using python scripts, called the convex hull of the TAD. The volume of each convex hull was calculated as the raw volume of the TAD.

### TAD Volume Normalized by Theoretical Random Walk

First, the TAD volume was calculated based on the hic matrix (Experimental Section:’ TAD volume calculation’). The theoretical random walk was calculated volume based on the length of TADs (we controlled the consistent volume of random walks in genomic TADs of equal length) and normalized the original volume by dividing it by the theoretical random walk volume. This normalization was performed to account the influence of TAD length for the TAD volume. Specifically, the research methodology consists of three steps: 1. Initializing the polymer chain model. 2. Performing random walk simulations. 3. Calculating volumes. Here, a chromatin persistence length of 50 nm was adopted, consistent with Justin P. Peters et al,^[^ ^65^ ^]^ Catherine Tardin et al.^[^ ^66^ ^]^ Then, the normalized volume can be used for calculating TCI (the original volume dividing by the theoretical random walk).

### TCI Calculation

A TCI was proposed to characterize the interaction density within each TAD, the raw TCI was calculated as the original volume dividing by the theoretical random walk volume. Since the length of TAD may be affected by the change of sequence depth or the adoption of different call TAD method algorithms, a quantile normalization used the preprocessCore package in R for each original TCI. The normalized TCI was classified into four categories according to the threshold (‐1, 0, 1) for all TADs. The normalized TADs have a consistent distribution, which facilitates a more refined comparison between different cell line datasets in subsequent analyses. A smaller TCI indicates that the TAD was more compact than expected in a random conformation, indicating a greater density of chromatin interactions within the TAD

### Validation of the TCI

To ensure that the reconstructed structures and volumes accurately reflect the 3D characteristics of chromatin, including TAD length and interaction patterns, a rigorous validation of the TCI metric was conducted. This involved assessing both the reliability of TAD volume calculations and the impact of TAD length on TAD volume, thereby reinforcing the robustness and authenticity of the approach.

### Dependability of TAD Volume Calculation

We substantiate the dependability of TAD volume calculation was substantiated through the following aspects:

(i) Employing FLAMINGO, a high‐resolution reconstruction method that has already been validated for optimal performance across various contexts, including microscopy chromatin image‐based tracking data, original Hi‐C, and simulated data.

(ii) Utilizing FLAMINGO for the 3D reconstruction of TAD coordinates, TAD volume was measured by constructing convex hulls based on the 3D coordinates. Specifically, the convex hull of TADs was formed using a set of points derived from 3D spatial coordinates, and quantify the volume of each TAD by calculating its convex hull. This method of quantifying chromatin structure volume using convex hull calculations was consistent with approaches adopted in previous studies.^[^ ^67^ ^]^

(iii) Using single‐cell imaging data as measured by Xiaowei Zhuang et al,^[^ ^60^ ^]^ FLAMINGO was employed to compute the volumes of genuine single‐cell chromatin segments. The calculated volumes reflect the differences in the 3D space occupied by chromatin regions tracked via single‐cell imaging. For example, the region contacts in IMR90 Cell15 were notably higher than in Cell12 (Figure S10B, Supporting Information), and the results indicated a smaller volume for Cell15 (Figure S10B, Supporting Information). Furthermore, the average Euclidean distance (Experimental Section) of loci within the single‐cell region suggested that Cell15 had the shortest distance, indicating that stronger contacts correspond to a more compact volume, consistent with previous findings (Figure S10B, Supporting Information).

(iv) Applying the convex hull calculation method to quantitatively analyze the volume of chromatin structures, the correlation was evaluated between the real volume of chromatin tracking data provided by high‐resolution imaging^[^ ^60^ ^]^ (Xiaowei Zhuang et al.) and the volume obtained by four reconstruction methods. The analysis showed that the volume reconstructed by FLAMINGO had the highest correlation with the actual volume (Figure S10C, Supporting Information), indicating that FLAMINGO accurately reflects the differences in volume between different cells.

### Excluding the Influence of TAD Length on TAD Volume

Given that TAD coordinates were reconstructed from the input Hi‐C interaction matrix, different lengths of TADs imply varying numbers of interactions. To address this issue, the influence of TAD length on TAD volume was excluded.

(i): Median Contact Frequency Scaling (MCFS) correction was performed on the Hi‐C matrix (Experimental Section). MCFS normalization, facilitated by gcMapExplorer (https://gcmapexplorer.readthedocs.io/en/latest/cmapNormalization.html) standardizes contact maps to eliminate biases due to genomic distance. This approach aims to normalize contact frequencies at different distances. The median contact value normalizes the Hi‐C contact map based on the distance between two genomic loci. Initially, the expected contact frequency (median or average) for each specific distance was calculated. Subsequently, the observed contact frequency was either divided by or subtracted from the expected value for the corresponding distance to achieve normalization. The objective was to bring the observed interaction frequencies in each region closer to their expected values, thereby reducing or eliminating the influence of interaction frequency on linear genomic distance.

(ii): To elucidate the relationship between TAD volume and interactions while controlling for TAD length, TADs were stratified by length. For each class of TAD lengths, if the number of TADs exceeded five, Z‐score normalization was performed on this class of TADs. Thus, the normalized TAD volume represents the relative volume of that TAD compared to others of the same length. A low Z‐score normalized volume (≤ ‐0.5) and a high Z‐score normalized volume (≥ 0.5) were designated. The results indicated that smaller TADs exhibited higher interaction frequencies compared to larger TADs (Figure S10D, top, Supporting Information). These findings were consistent with previous studies, suggesting that simulated TAD volumes correspond to the interaction patterns of real TADs. Further analysis showed that these smaller TADs had a higher susceptibility to DSBs (Figure S10D, bottom, Supporting Information), corroborating the results of subsequent analysis.

(iii): The theoretical random walk volume was calculated based on the length of TADs and then normalized the original volume by dividing it by the theoretical random walk volume to derive the TCI (Experimental Section). This normalization was performed to account for the influence of TAD length. Since DNA fragments of different lengths naturally possess different volumes, this difference due to length can be mitigated by dividing by the theoretical random walk volume. A smaller ratio indicates a more compact conformation of the TAD. The compactness of different TCI TADs was demonstrated using the average Euclidean distance (Experimental Section), and the TADs with the lowest TCI (Level 1) correspond to closer spatial distances (Figure S11A, Supporting Information).

### Calculate Pairwise TAD contact

Here is a detailed description of the calculation process for normalized pairwise TAD‐TAD contact:

Step1: Calculate the raw pairwise TAD contact frequency (Cij) for two TADs i and j:

(13) $$C_{ij}=\frac{n}{\left(L_{i}*L_{j}\right)}$$

Where *n* is the total sum of contact frequency between genomic regions of the two TADs. *L_i_*, *L_j_* represent the length of two TADs.

Step2: Calculate the genomic distance between TAD pairs as the distance between the midpoints of the two TADs.

Step3: Perform loess regression of the pairwise TAD contact frequencies against the genomic distances between TAD pairs. This gives the expected contact frequency (µ_*d*_) for each distance.

Step4:Calculate the standard deviation of pairwise TAD contact frequency at each distance. Perform loess regression of these standard deviations against genomic distance. This gives the expected standard deviation (σ_*d*_) for each distance.

Step5:Calculate the normalized pairwise TAD contact frequency:

(14) $$TAD_{ij}=\frac{C_{ij}-\mu_{d}}{\sigma_{d}}$$

Where *C_ij_* is the raw contact frequency (from Step1), µ_*d*_ is the expected contact frequency at that distance (from Step3), σ_*d*_ is the expected standard deviation at that distance (from Step4). The resulting *TAD_ij_* are the normalized pairwise TAD‐TAD contact frequencies.

### Calculate the HubTADs

For TADs in the genome, they were classified based on the extent of their overlap with hub bins. Taking the calculation of hubTADs at 10Kb resolution as an example, to maximize the characteristics of hub nodes, a TAD was defined as one that must contain at least 10 or more hub nodes. The parameter bedops ‐e 10 000 was used to determine the hubTADs. The other TADs were calculated in the same manner.

### Data Processing for DSB Formation

DSB and corresponding Hi‐C data from U2OS cell lines before and after OHT (4‐hydroxytamoxifen) induction^[^ ^30^ ^]^ (Accession number E‐MTAB‐8851) were collected. The changes in TADs before and after OHT induction were calculated. From TADs with unchanged length before and after OHT induction (to ensure that they were the same TAD), TADs without DSB before OHT induction (that is, DSB‐free TADs with no DSB occurrence) and an increased DSB after OHT induction (that is, DSB TADs with DSB occurrence) were screened out. In NHEK cell line. TADs of the same length were classified into those without DSBs and those with DSBs based on the number of DSBs. In the breast cancer development model from MCF10A to MCF7 cell lines, a similar operation was performed. Specifically, TADs of consistent length throughout the development from MCF10A to MCF7 were selected (to control for the same TAD) and then classified these TADs based on their DSB numbers: TADs without DSBs in MCF10A (GSE93040) and TADs with DSBs in MCF7.

### RNA‐seq Data Processing

The breast cancer (BRCA: Breast invasive carcinoma) RNA‐seq data (FPKM) was downloaded from the Cancer Genome Atlas (TCGA) project collected by XENA (https://gdc.xenahubs.net). Then, limma^[^ ^68^ ^]^ was used to perform differential gene expression analysis. Log_2_ fold change > 1 and *p*‐value < 0.05 were used as cutoff to screen Up‐regulated genes, and log_2_ fold change < ‐1 and p‐value < 0.05 were used as cutoff to screen Down‐regulated genes. Meanwhile, R package “survival” was used to perform univariate independent prognostic analysis. And colorectal cancer survival was analyzed using the “survminer” R package. (*P* < 0.01 was considered significant).

### Experimental Procedures for siRNA Transfection and mRNA Quantification

MCF‐7 cells were obtained from the National Collection of Authenticated Cell Cultures. siRNA and DNA oligos were purchased from Sangon Biotech. MCF‐7 cells were cultured in DMEM/F12k medium supplemented with 10% fetal bovine serum (FBS) and 2 mm GlutaMAX at 37 °C under 5% CO2. For siRNA knockdown experiments, cells were seeded in 12‐well plates. When the cells reached ≈80% confluence, they were trypsinized and resuspended in a complete medium for cell counting, then diluted to a concentration of 1 × 10^6 cells mL^−1^. The siRNA transfection procedure followed the protocol outlined in the Lipofectamine RNAiMAX manual for MCF‐7 cells, using reagents as specified by the starting point quantities.After 48 h of culture, cells were trypsinized and resuspended in 120 µL of complete medium for cell counting. Total RNA was extracted using Trizol, and 90 ng of RNA was used for reverse transcription following the protocol provided by the SuperScript IV First‐Strand Synthesis System. One microliter of the reverse transcription product was used for qPCR validation, which was performed according to the KAPA SYBR® FAST qPCR Master Mix (2X) Kit instructions. GAPDH served as the internal reference for normalizing qPCR data. The primer sequences used in this study are provided in (Table S8, Supporting Information).

### Spatial Organization Analysis of Five Risk Genes within hubTADs

To explore their (*ESRP1, GRHL2, MTHFD2, RACGAP1002C, and SQLE*) spatial organization, the hubTADs containing these five genes were analyzed. Within each hubTAD, the chromatin interaction frequencies between bins harboring the five risk genes (candidate gene loci) and other chromatin nodes, including both hub and non‐hub nodes were evaluated. Furthermore, the candidate gene loci and other chromatin nodes were used to calculate the Euclidean distances based on Hi‐C data in hubTADs.

### Statistical Analysis

Data Pre‐processing: All data preprocessing methods were described in detail in the Experimental Section. Specifically, for Hi‐C data, the MCFS (Multi‐Contact Frequency Smoothing) normalization was applied to correct for sequencing biases and technical noise. For 3D coordinate data, centering and F‐norm normalization were performed to ensure comparability across samples. These preprocessing steps provide a solid foundation for downstream analyses.

Data Presentation: Detailed descriptions of the data presentation formats can be found in the main text and Supporting Information. For example, to evaluate the performance stability of Hi‐DSB, mean ± standard deviation (mean ± SD) was used as a quantitative measure across repeated experiments or simulations. For other comparative or genomic feature‐related analyses, log2 transformation was applied to the data to normalize distributions and enhance visualization clarity. This allowed us to better capture relative changes and improve statistical interpretability.

Sample Size: The sample size for the analysis was explicitly indicated in the corresponding figure legends or main text.

Statistical Methods: For statistical methods, unless otherwise specified, the Wilcoxon test was used to assess significant differences. The significance threshold was set at p = 0.05. Detailed descriptions of these analyses can be found in the main text or figure legends.

Software: The software tools used for the analyses are described in the Experimental Section. Unless otherwise specified, all computational analyses were performed using Python (v3.12.4).

Human gene expression (RNA‐seq) data were downloaded from GEO dataset under accession numbers GSE78594 (NHEK), GSM2343891 (K562), and GSM2400218 (MCF7).

Data of super‐enhancers in the NHEK, K562, and MCF7 cell lines were downloaded from http://www.licpathway.net/sedb.

Hi‐C data were downloaded from the GEO dataset under accession numbers GSE63525 (NHEK), GSE63525 (K562), GSM2935613 (MCF7), GSE133928 (HCT116), GSE93040 (MCF10A). Annotations of chromatin loops for human cell lines (NHEK, MCF7, K562) were downloaded from Yue Feng lab.^[^ ^69^ ^]^

ChIP‐seq and DNase‐seq for human cell lines (NHEK, MCF7, K562) could be found in Mourad et al^[^ ^20^ ^]^ from ENCODE.^[^ ^70^ ^]^ ChIP‐seq for HCT116 would be downloaded from GEO dataset under accession numbers GSM2916001 (H3K27ac), GSM945304 (H3K4me3), GSE231292 (CTCF). DNase‐seq for HCT116 would be downloaded from GEO dataset under accession number GSM736600.

All double‐strand DNA break data used are summarized in the (Table S9, Supporting Information). Briefly, double‐strand DNA breaks mapped by DSBCapture and BLESS in NHEK cell line under GEO accession GSE78172; BLISS in K562 and MCF7 cell line was available on SRA with accession SRP150605; END‐seq in Nalm6 cell lines under GEO accession GSE99197; END‐seq in HCT116 cell lines under GEO accession GSE129529 were used.

## Conflict of Interest

The authors declare no conflict of interest.

## Author Contributions

K.X., Z.Y., C.S. contributed equally to this work. X.B., H.C., H.L., and G.Y. conceived this study; H.C. provided systematic guidance on 3D genome exploration; K.X., Z.Y., and S.C. implemented the algorithm and analyzed the data; K.X., Z.Y., and S.C. designed the model; C.G., J.Z, and J.W. assisted with the implementation of the study and data analysis; K.X. wrote the paper.

## Supporting information



Supporting Information

Supporting Information

## Data Availability

We collected data from public datasets and did not generate new data. These data can be found in the article. The source codes for running Hi‐DSB and TCI are available on GitHub (https://github.com/xk‐idea/Hi_DSB).
