# Supplementary material for: ChromInSight: Revealing DNA Double‐Strand Breaks Through Chromatin Structural Insights With an Interpretable Graph Neural Network Framework
Source: Adv Sci (Weinh). 2025 Jun 30;12(36):e04571. doi: 10.1002/advs.202504571 (PMC12463085; doi:10.1002/advs.202504571)
Supplement: Supplementary file 1 — Supporting Information [file ADVS-12-e04571-s001.pdf]

ADVANCED SCIENCE
Open Access

Supporting Information

for *Adv. Sci.*, DOI 10.1002/advs.202504571

ChromInSight: Revealing DNA Double-Strand Breaks Through Chromatin Structural Insights With an Interpretable Graph Neural Network Framework

Kang Xu, Zongyuan Yu, Canzhuang Sun, Conglin Gou, Jiangyue Zhu, Jun Wang, Xiaochen Bo, Guoxian Yu*, Hao Li* and Hebing Chen**

Supporting Information

FigS1:

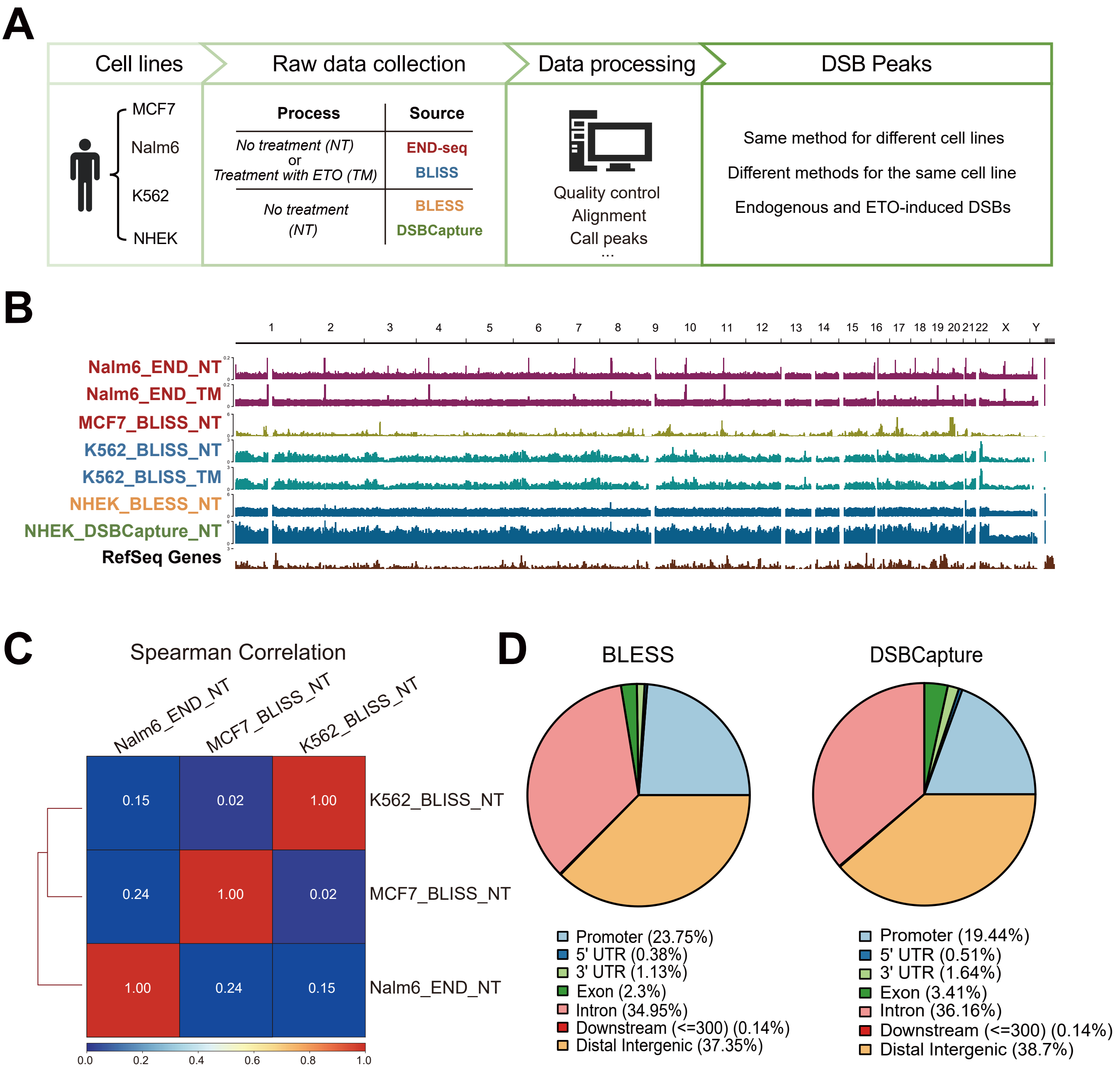

Figure S1. Multifaceted comparison and development standardized DSB dataset. (**A**) Schematic flow chart of DSB raw data processing. (**B**) Genome-wide distribution of endogenous (No Treatment, NT) and ETO-induced (Treatment, TM) DSBs generated by different methods in different cell lines. (**C**) Cross correlations from various cell types (MCF7, K562, Nalm6) were calculated using bins of 10kb. Results demonstrate low correlations among different cell types. (**D**) Distribution of DSB detected by two methods (BLESS, DSBCapture) across the genome.

FigS2:

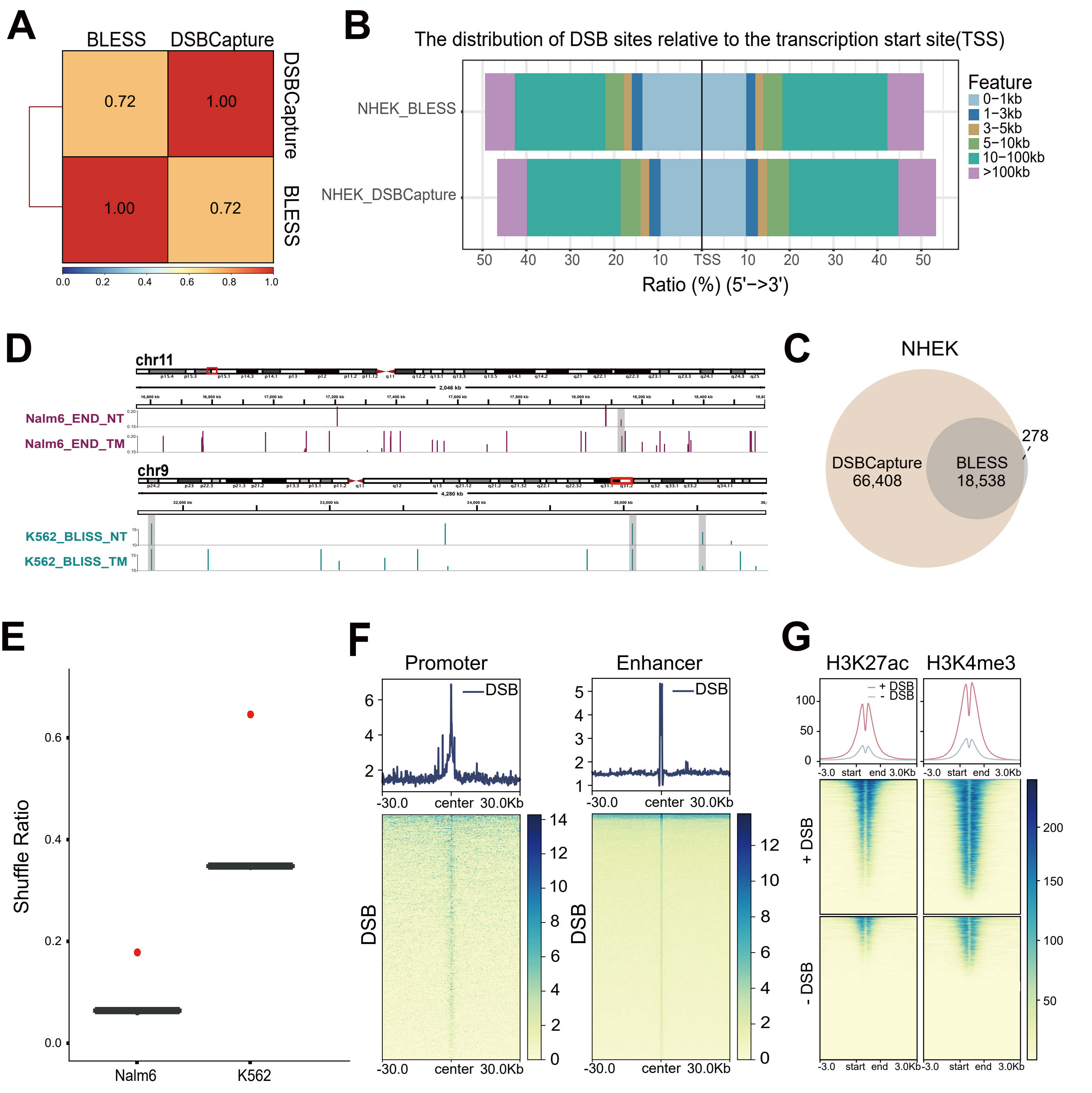
A
BLESS
DSBCapture
0.72
1.00
1.00
0.72
0.0 0.2 0.4 0.6 0.8 1.0
B
The distribution of DSB sites relative to the transcription start site(TSS)
NHEK_BLESS
NHEK_DSBCapture
50 40 30 20 10 TSS 10 20 30 40 50
Ratio (%) (5'->3')
Feature
0−1kb
1−3kb
3−5kb
5−10kb
10−100kb
>100kb
D
chr11
Nalm6_END_NT
Nalm6_END_TM
chr9
K562_BLISS_NT
K562_BLISS_TM
C
NHEK
DSBCapture
66,408
BLESS
18,538
278
E
Shuffle Ratio
0.0
0.2
0.4
0.6
Nalm6
K562
F
Promoter
Enhancer
DSB
-30.0
center
30.0Kb
G
H3K27ac
H3K4me3
+ DSB
- DSB
-3.0
start
end
3.0Kb

Figure S2. Comparison of DSBs. (**A**) Cross correlations (Spearman) between samples of the same cell line (NHEK) using different methods (BLESS, DSBCapture). (**B**) The panel illustrates the distribution of DSB sites relative to the transcription start site (TSS) of the nearest gene. (**C**) Comparison of DSBs obtained using different methods (DSBCapture, BLESS) for the same cell line (NHEK). (**D**) Genome browser view of the same and different genomic regions in endogenous and ETO-induced DSBs. (**E**) The proportion of ETO-induced DSBs within endogenous DSBs; red dot represent the true ratio of ETO-induced DSBs within endogenous DSBs, compared to the ratio of 100 random regions of the same length as the endogenous DSBs. $P < 0.01$. (**F**) Enrichment distribution of DSBs at promoters and enhancers in MCF7 cell line. Top: Average distribution of DSBs within ±30 kb of promoters and enhancers. Bottom: Heatmaps centered on promoters and enhancers. The color bar represents the intensity range based on DSB enrichment, with yellow to blue representing low to high enrichment values. (**G**) DSB-containing (+DSB) promoters are enriched for histone modification H3K27ac and H3K4me3 signals in MCF7 cell line, compared to DSB-free (-DSB) promoters

FigS3:

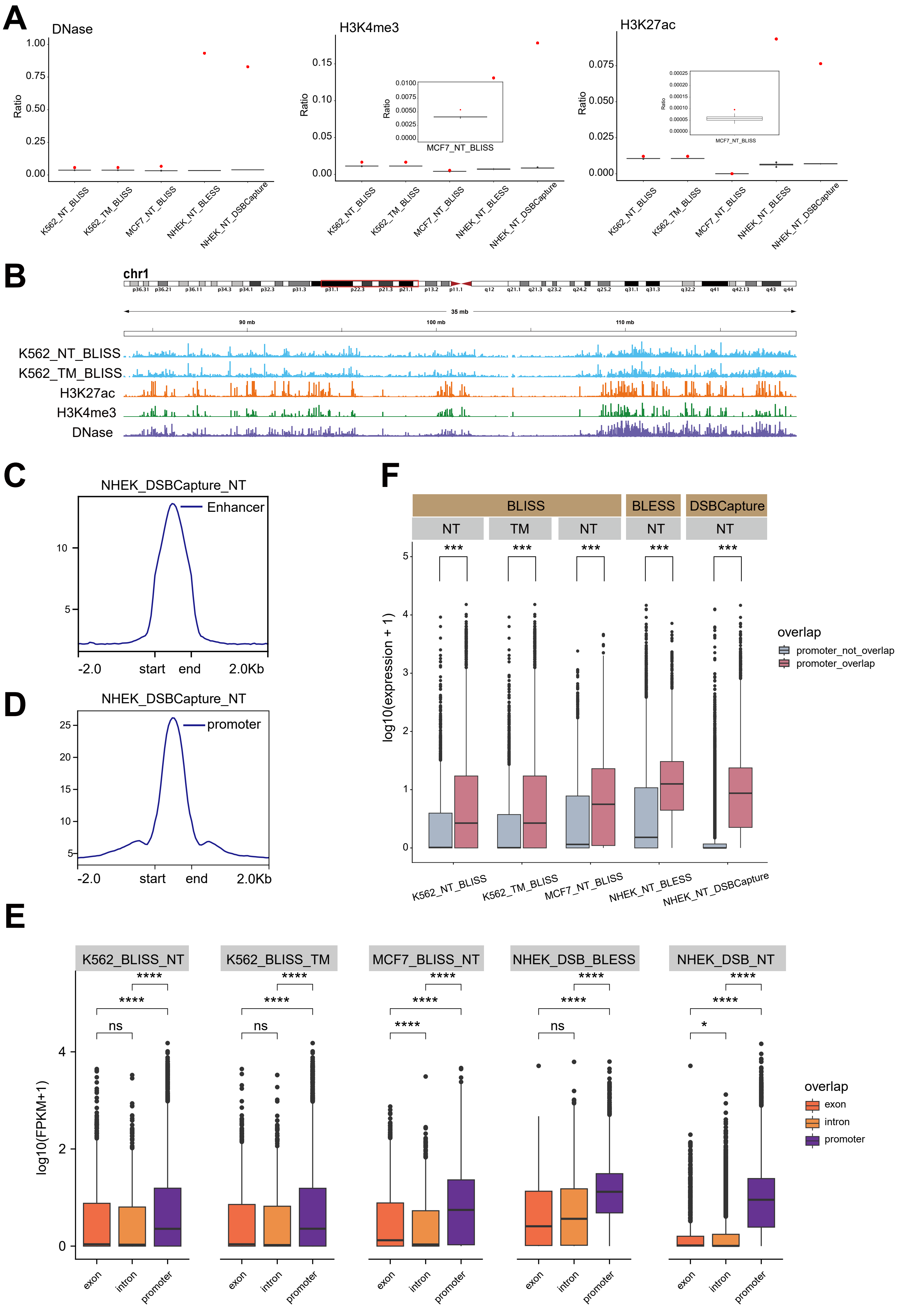
A
DNase
Ratio
1.00
0.75
0.50
0.25
0.00
K562_NT_BLISS
K562_TM_BLISS
MCF7_NT_BLISS
NHEK_NT_BLESS
NHEK_NT_DSBCapture
H3K4me3
0.15
0.10
0.05
0.00
0.0100
0.0075
0.0050
0.0025
0.0000
MCF7_NT_BLISS
H3K27ac
0.075
0.050
0.025
0.000
0.00025
0.00020
0.00015
0.00010
0.00005
0.00000
B
chr1
35 mb
90 mb
100 mb
110 mb
K562_NT_BLISS
K562_TM_BLISS
H3K27ac
H3K4me3
DNase
C
NHEK_DSBCapture_NT
Enhancer
10
5
-2.0
start
end
2.0Kb
D
NHEK_DSBCapture_NT
promoter
25
20
15
10
5
F
BLISS
BLESS
DSBCapture
NT
TM
***
log10(expression + 1)
overlap
promoter_not_overlap
promoter_overlap
E
K562_BLISS_NT
K562_BLISS_TM
MCF7_BLISS_NT
NHEK_DSB_BLESS
NHEK_DSB_NT
****
ns
*
log10(FPKM+1)
4
2
0
exon
intron
promoter

Figure S3. Genomic characterization of DSBs. (**A**) Ratio of H3K27ac, H3K4me3, and DNase enriched in DSBs of different cell lines by different methods; red triangles represent the true ratio of histone modifications enriched in DSBs, compared to the ratio of 1000 random regions of the same length as the DSBs. $P < 0.001$. (**B**) Genome browser showing clear colocalization of endogenous and ETO-induced DSBs with active histone modifications H3K27ac, H3K4me3 and DNase signal in K562 cell line. (**C, D**) Average distribution of DSBs within ±2 kb of promoters and enhancers in NHEK cell line. (**E**) Boxplots compare the gene expression with DSBs located at exons, introns, and promoters of genes, respectively. Upper brown boxes indicate different methods, grey boxes indicate DSBs are endogenous (NT) or ETO induced (TM). *$P < 0.05$, **$P < 0.01$, ***$P < 0.001$, ****$P < 0.0001$, Wilcox test. (**F**) Boxplots showing comparison of the expression levels of gene with and without DSB at promoters in four cell lines (MCF7, K562, NHEK). ****$P < 0.0001$, Wilcox test.

FigS4:

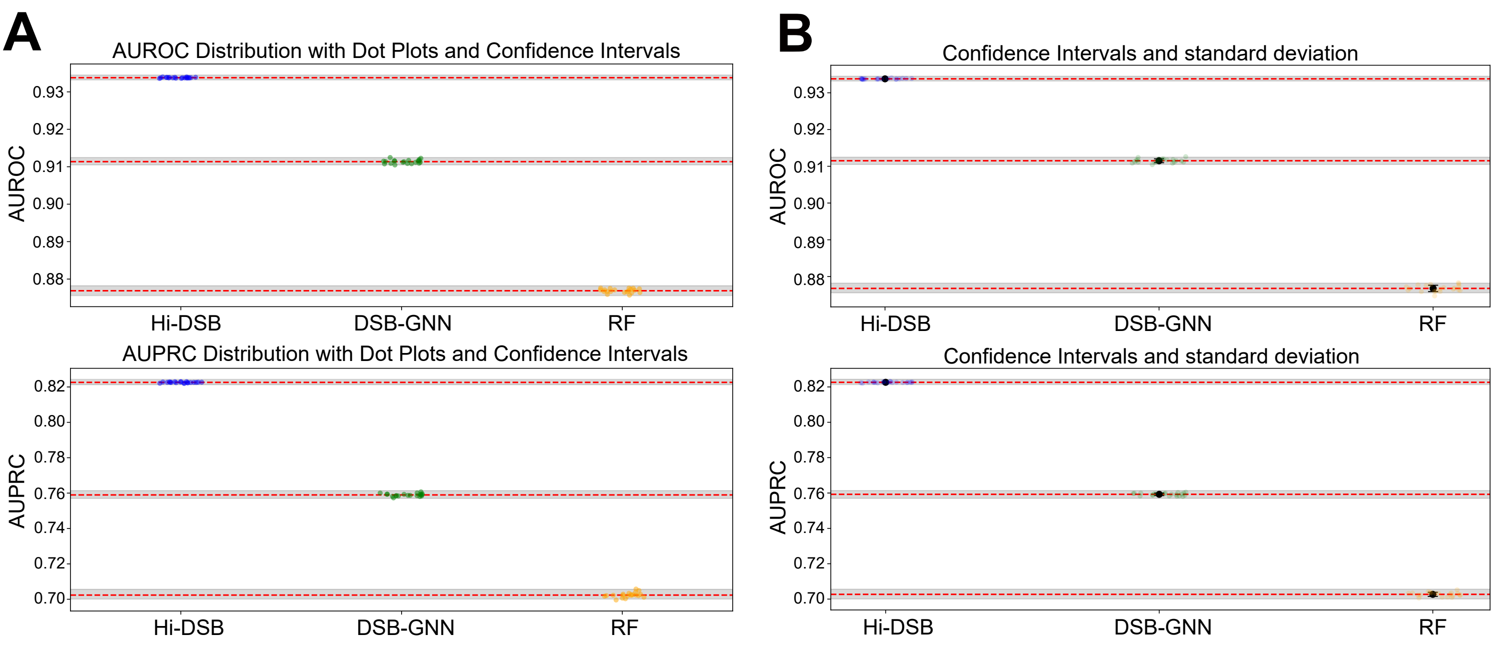

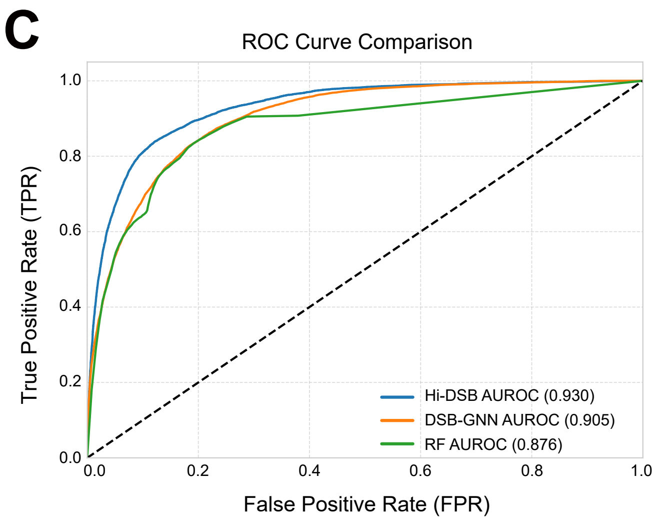

D

Method1	Method2	DeLong (P-Value)
Hi-DSB	DSB-GNN	1.486e-168
Hi-DSB	RF	2.442e-161
DSB-GNN	RF	2.115e-31

Figure S4. Comparative assessment of Hi-DSB, DSB-GNN, and RF. (**A**) AUROC and AUPRC Distribution with Dot Plots and Confidence Intervals, the red dashed lines represent the mean performance of each model, while the grey shaded regions indicate the 95% confidence intervals. (**B**) AUROC and AUPRC Distribution with Dot Plots and Confidence Intervals with error bars representing ± standard deviation. (**C**) Compares the receiver operating characteristic (ROC) curves of three methods: Hi-DSB, DSB-GNN, and RF. The x-axis represents the false positive rate (FPR), and the y-axis represents the true positive rate (TPR). The area under the curve (AUC) values are as follows: Hi-DSB (0.930), DSB-GNN (0.905), and RF (0.876). (**D**) The DeLong test to evaluate the statistical significance of the differences in AUC values.

FigS5:

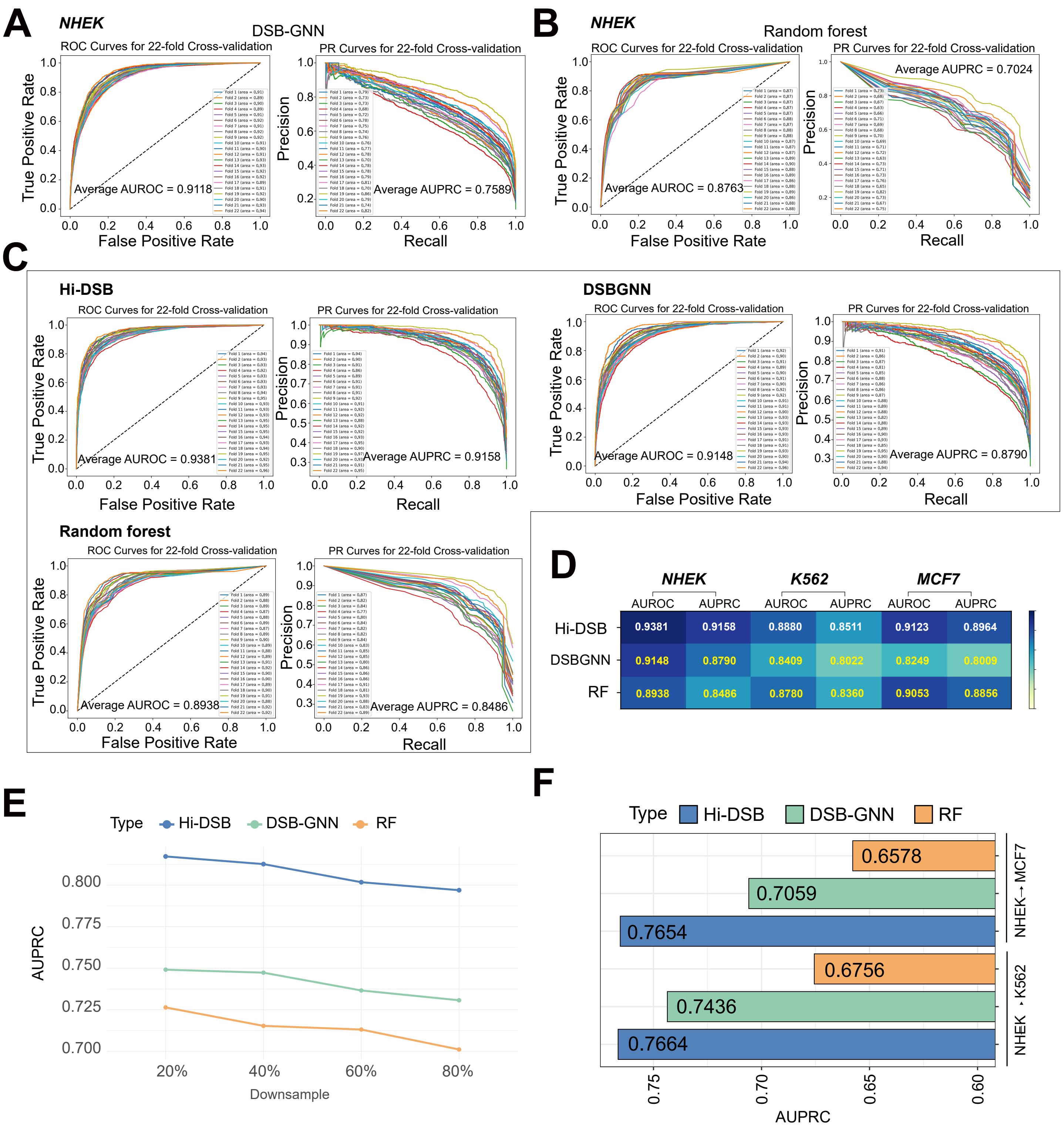
A
NHEK
DSB-GNN
ROC Curves for 22-fold Cross-validation
True Positive Rate
False Positive Rate
Average AUROC = 0.9118
PR Curves for 22-fold Cross-validation
Precision
Recall
Average AUPRC = 0.7589
B
NHEK
Random forest
ROC Curves for 22-fold Cross-validation
True Positive Rate
False Positive Rate
Average AUROC = 0.8763
PR Curves for 22-fold Cross-validation
Precision
Recall
Average AUPRC = 0.7024
C
Hi-DSB
ROC Curves for 22-fold Cross-validation
True Positive Rate
False Positive Rate
Average AUROC = 0.9381
PR Curves for 22-fold Cross-validation
Precision
Recall
Average AUPRC = 0.9158
DSBGNN
ROC Curves for 22-fold Cross-validation
True Positive Rate
False Positive Rate
Average AUROC = 0.9148
PR Curves for 22-fold Cross-validation
Precision
Recall
Average AUPRC = 0.8790
Random forest
ROC Curves for 22-fold Cross-validation
True Positive Rate
False Positive Rate
Average AUROC = 0.8938
PR Curves for 22-fold Cross-validation
Precision
Recall
Average AUPRC = 0.8486
D
NHEK
K562
MCF7
AUROC
AUPRC
AUROC
AUPRC
AUROC
AUPRC
Hi-DSB 0.9381 0.9158 0.8880 0.8511 0.9123 0.8964
DSBGNN 0.9148 0.8790 0.8409 0.8022 0.8249 0.8009
RF 0.8938 0.8486 0.8780 0.8360 0.9053 0.8856
E
Type
Hi-DSB
DSB-GNN
RF
AUPRC
0.800
0.775
0.750
0.725
0.700
20%
40%
60%
80%
Downsample
F
Type
Hi-DSB
DSB-GNN
RF
0.6578
0.7059
0.7654
0.6756
0.7436
0.7664
NHEK→MCF7
NHEK→K562
0.75
0.70
0.65
0.60
AUPRC

Figure S5. Hi-DSB performance evaluation. (**A, B**) Performance Characteristics of (DSB-GNN, Random forest) in AUROC and AUPRC. To avoid overfitting, we conducted a 22-fold cross-validation, where each chromosome was sequentially used as an independent test set, while the remaining chromosomes formed the training set at 10kb resolution. (**C, D**) The average performance of three DSB prediction methods across different cell lines in 22-fold cross-validation is shown, with white highlighting the superior performance of Hi-DSB across different cell lines at 25kb resolution. (**E**) Impact of Downsampling Hi-C data on model performance (AUPRC). (**F**) Evaluate the model's ability to transfer from one cell line (NHEK) to two additional cell lines (K562 and MCF7) (AUPRC).

FigS6:

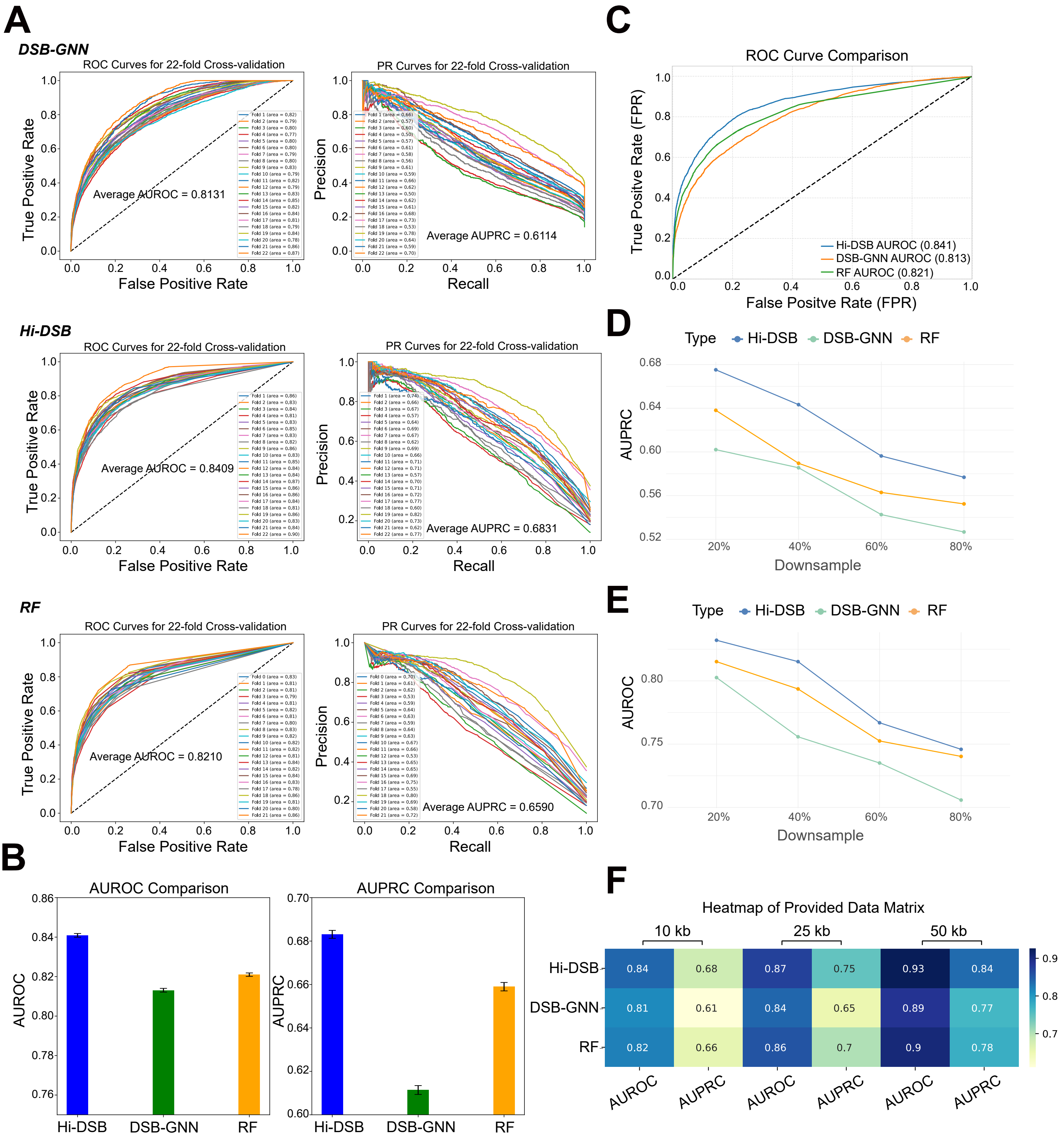
A
DSB-GNN
ROC Curves for 22-fold Cross-validation
Average AUROC = 0.8131
True Positive Rate
False Positive Rate
PR Curves for 22-fold Cross-validation
Average AUPRC = 0.6114
Precision
Recall
Hi-DSB
ROC Curves for 22-fold Cross-validation
Average AUROC = 0.8409
True Positive Rate
False Positive Rate
PR Curves for 22-fold Cross-validation
Average AUPRC = 0.6831
Precision
Recall
RF
ROC Curves for 22-fold Cross-validation
Average AUROC = 0.8210
True Positive Rate
False Positive Rate
PR Curves for 22-fold Cross-validation
Average AUPRC = 0.6590
Precision
Recall
B
AUROC Comparison
AUROC
Hi-DSB
DSB-GNN
RF
AUPRC Comparison
AUPRC
Hi-DSB
DSB-GNN
RF
C
ROC Curve Comparison
True Positive Rate (FPR)
False Positve Rate (FPR)
Hi-DSB AUROC (0.841)
DSB-GNN AUROC (0.813)
RF AUROC (0.821)
D
Type Hi-DSB DSB-GNN RF
AUPRC
Downsample
20% 40% 60% 80%
E
Type Hi-DSB DSB-GNN RF
AUROC
Downsample
20% 40% 60% 80%
F
Heatmap of Provided Data Matrix
10 kb 25 kb 50 kb
Hi-DSB 0.84 0.68 0.87 0.75 0.93 0.84
DSB-GNN 0.81 0.61 0.84 0.65 0.89 0.77
RF 0.82 0.66 0.86 0.7 0.9 0.78
AUROC AUPRC AUROC AUPRC AUROC AUPRC

Figure S6. Evaluation of model performance in HCT116. (**A**) Performance Characteristics of (DSB-GNN, Random forest) in AUROC and AUPRC. To avoid overfitting, we conducted a 22-fold cross-validation, where each chromosome was sequentially used as an independent test set, while the remaining chromosomes formed the training set at 10kb resolution. (**B**) The bar chart summarizes the average performance across the 22 folds, along with confidence intervals. (**C**) Compares the receiver operating characteristic (ROC) curves of three methods: Hi-DSB, DSB-GNN, and RF. The x-axis represents the false positive rate (FPR), and the y-axis represents the true positive rate (TPR). (**D, E**) Impact of Downsampling Hi-C data on model performance (AUPRC, AUROC). (**F**) Heatmap showing the performance of different models at various Hi-C resolutions.

FigS7:

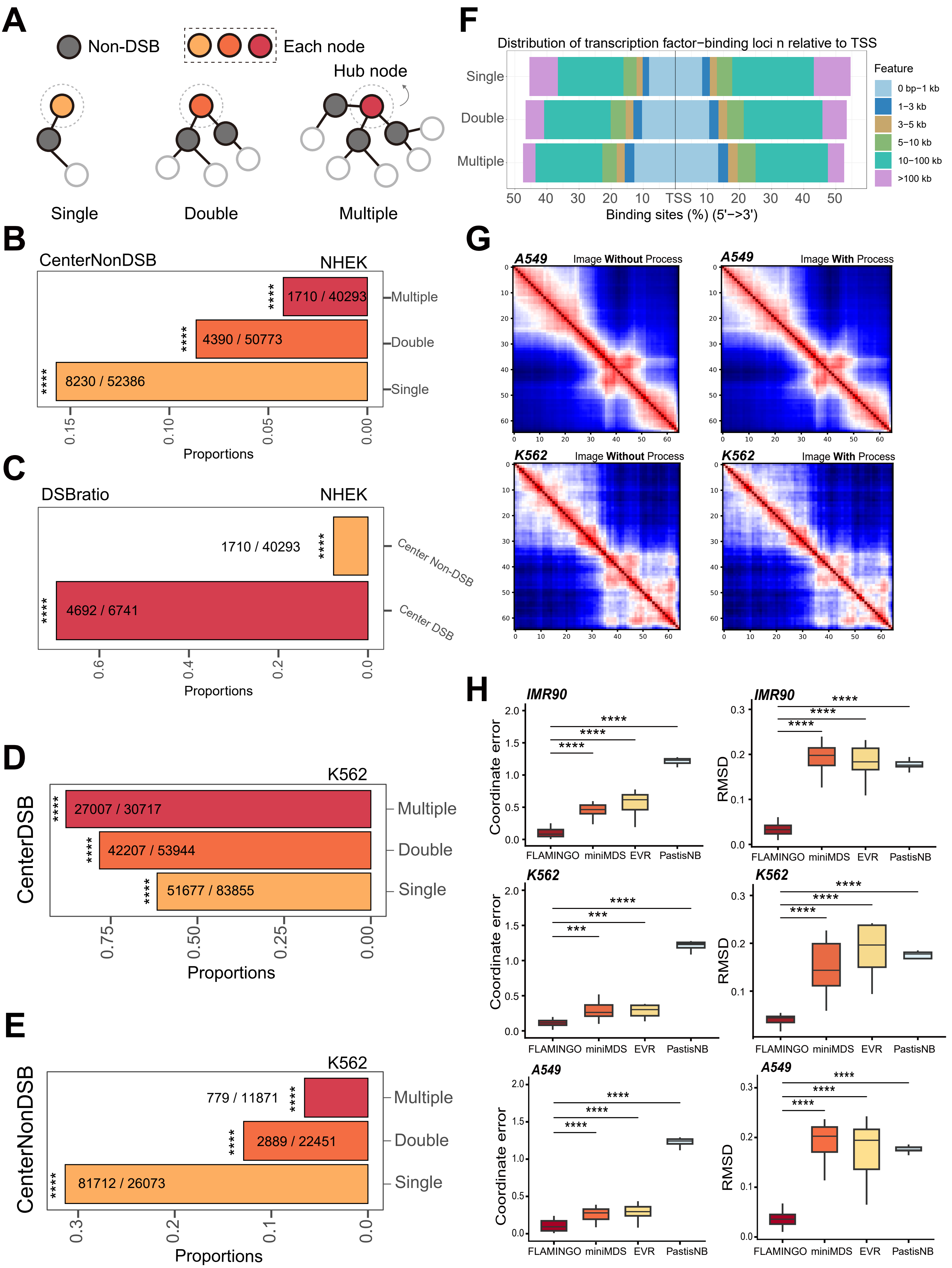
A
Non-DSB
Each node
Hub node
Single
Double
Multiple
B
CenterNonDSB
NHEK
1710 / 40293
4390 / 50773
8230 / 52386
Multiple
Double
Single
Proportions
C
DSBratio
NHEK
1710 / 40293
4692 / 6741
Center Non-DSB
Center DSB
Proportions
D
K562
CenterDSB
27007 / 30717
42207 / 53944
51677 / 83855
Multiple
Double
Single
Proportions
E
K562
CenterNonDSB
779 / 11871
2889 / 22451
81712 / 26073
Multiple
Double
Single
Proportions
F
Distribution of transcription factor−binding loci n relative to TSS
Single
Double
Multiple
Feature
0 bp−1 kb
1−3 kb
3−5 kb
5−10 kb
10−100 kb
>100 kb
Binding sites (%) (5'−>3')
G
A549
Image Without Process
A549
Image With Process
K562
Image Without Process
K562
Image With Process
H
IMR90
Coordinate error
RMSD
FLAMINGO miniMDS EVR PastisNB
K562
A549

Figure S7. Evaluation of high-resolution 3D reconstructed spatial model. (**A**) Schematic illustration of three patterns of connections between each node and Non-DSB sites (Number of single = 52386, double = 50773, multiple = 40293). (**B**) Probability of DSB occurrence under three different patterns of Non-DSB sites, **P < 0.01, *** P < 0.001, ****P < 0.0001, Chi-squared proportion test. (**C**) Probability of DSB occurrence at center DSB vs. center Non-DSB of Hub node, **P < 0.01, *** P < 0.001, ****P < 0.0001, Chi-squared proportion test. (**D**) Probability of DSB occurrence under three different patterns of DSB sites in K562 cell line, **P < 0.01, *** P < 0.001, ****P < 0.0001, Chi-squared proportion test. (**E**) Probability of DSB occurrence under three different patterns of Non-DSB sites in K562 cell line, **P < 0.01, *** P < 0.001, ****P < 0.0001, Chi-squared proportion test. (**F**) The panel illustrates the distribution of DSB sites relative to the transcription start site (TSS) of the nearest gene. (**G**) The comparison between the processed (linear interpolation, centering, and F-norm normalization) average distance map with the original average distance matrix (A549, K562). (**H**) RMSD and Coordinate error was employed to quantify the differences between the reconstructed three-dimensional chromatin structures using various methods and the structures obtained through microscopy imaging. **P < 0.01, *** P < 0.001, ****P < 0.0001, Wilcox test.

FigS8:

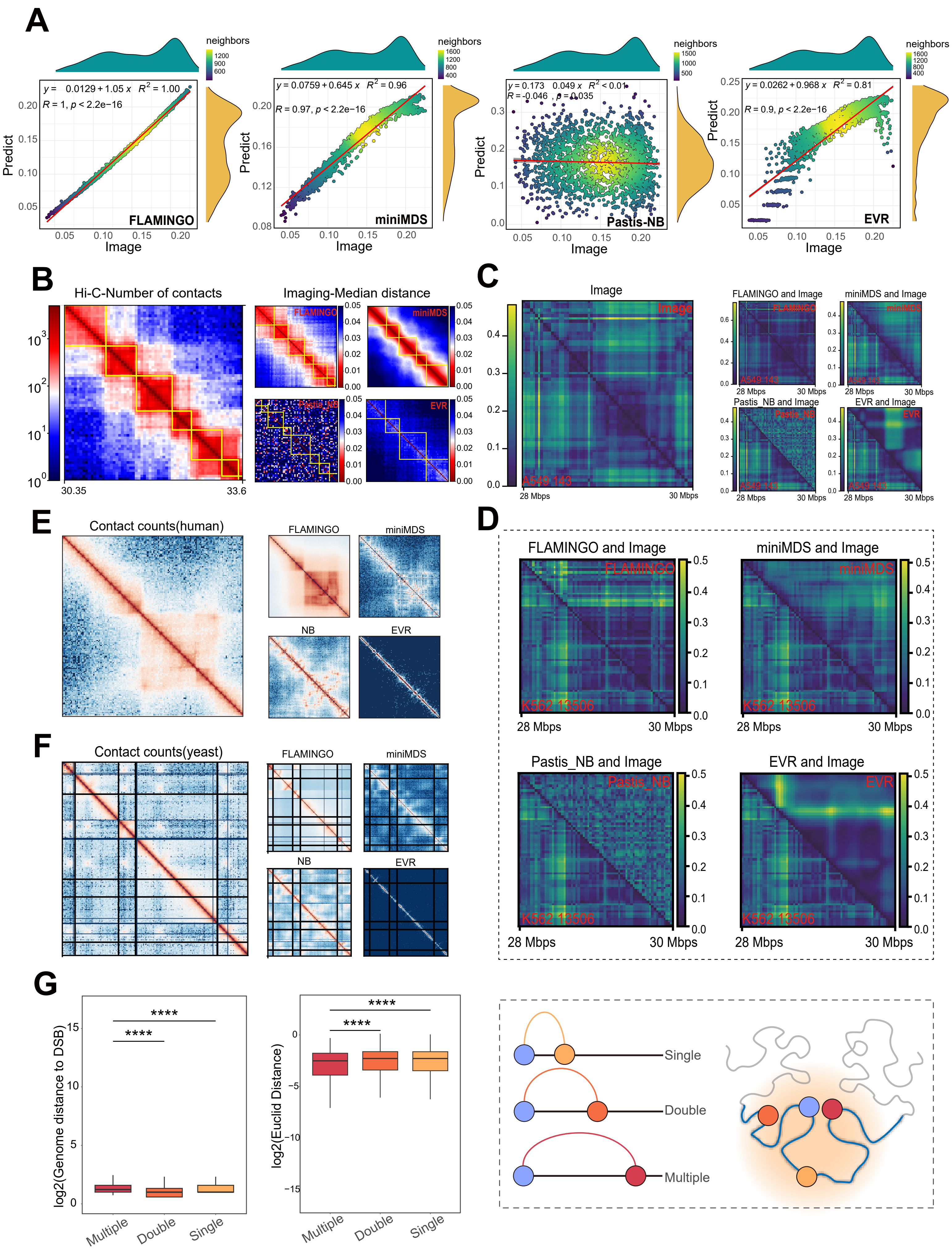

A
neighbors
$y = 0.0129 + 1.05\,x$ $R^2 = 1.00$
$R = 1$, $p < 2.2e{-16}$
FLAMINGO
$y = 0.0759 + 0.645\,x$ $R^2 = 0.96$
$R = 0.97$, $p < 2.2e{-16}$
miniMDS
$y = 0.173 - 0.049\,x$ $R^2 < 0.01$
$R = -0.046$, $p = 0.035$
Pastis-NB
$y = 0.0262 + 0.968\,x$ $R^2 = 0.81$
$R = 0.9$, $p < 2.2e{-16}$
EVR
Predict
Image
B
Hi-C-Number of contacts
Imaging-Median distance
30.35
33.6
FLAMINGO
miniMDS
Pastis_NB
EVR
C
Image
A549 143
28 Mbps
30 Mbps
FLAMINGO and Image
miniMDS and Image
Pastis_NB and Image
EVR and Image
D
FLAMINGO and Image
miniMDS and Image
Pastis_NB and Image
EVR and Image
K562 13506
28 Mbps
30 Mbps
E
Contact counts(human)
FLAMINGO
miniMDS
NB
EVR
F
Contact counts(yeast)
FLAMINGO
miniMDS
NB
EVR
G
log2(Genome distance to DSB)
log2(Euclid Distance)
****
Multiple
Double
Single

Figure S8. Evaluation of high-resolution 3D reconstructed spatial model. (**A**) Correlation analysis of cell average spatial distance matrices from four reconstructed models with super-resolution chromatin tracing data. (**B**) Comparison of median spatial distance matrices from Imaging with Integrated Hi-C contact matrices (chr21, GSE63525). (**C**) An illustration of the A549 143rd single cell 3D structure as depicted by four high-resolution reconstruction methods in diffraction-limited 3D imaging. (**D**) An illustration of the K562 13506rd single cell 3D structure as depicted by four high-resolution reconstruction methods in diffraction-limited 3D imaging. (**E**) The performance of four high-resolution reconstruction methods on the real Hi-C dataset of the GM12878 (GSE63525) cell line at 5KB resolution (chr21:30125000-30915000). (**F**) The performance of four high-resolution reconstruction methods on the real Hi-C dataset of yeast from `iced.datasets.load_sample_yeast()`. (**G**) The phenomenon where linearly distant - spatially proximal is also observed in K562 cell line.

FigS9:

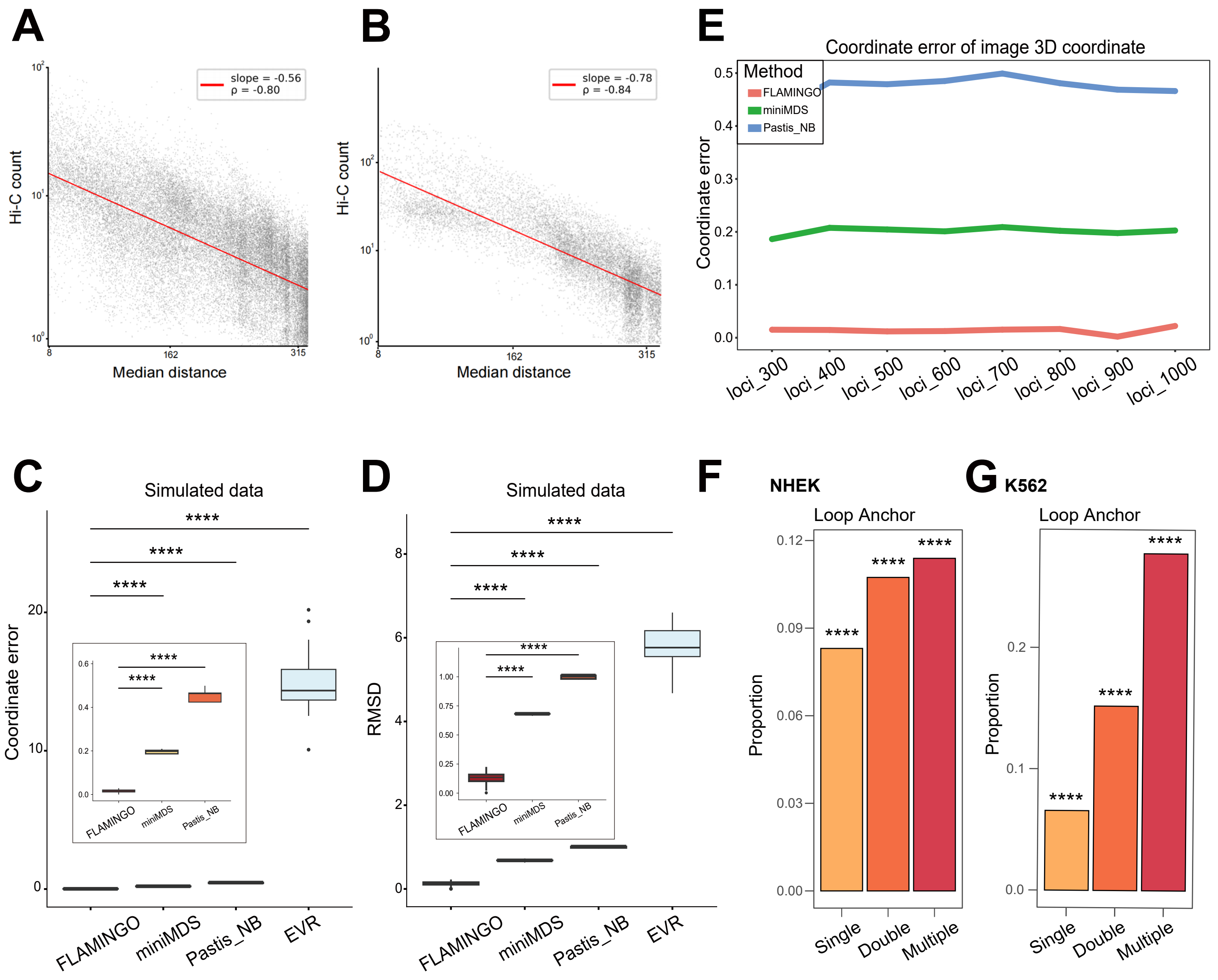

A
slope = -0.56
ρ = -0.80
Hi-C count
Median distance
B
slope = -0.78
ρ = -0.84
Hi-C count
Median distance
E
Coordinate error of image 3D coordinate
Method
FLAMINGO
miniMDS
Pastis_NB
Coordinate error
loci_300
loci_400
loci_500
loci_600
loci_700
loci_800
loci_900
loci_1000
C
Simulated data
Coordinate error
FLAMINGO
miniMDS
Pastis_NB
EVR
D
Simulated data
RMSD
FLAMINGO
miniMDS
Pastis_NB
EVR
F
NHEK
Loop Anchor
Proportion
Single
Double
Multiple
G
K562
Loop Anchor
Proportion
Single
Double
Multiple

Figure S9. **Evaluation of high-resolution 3D reconstructed spatial model.** (**A, B**) The scatter plot illustrates a significant negative correlation between the three-dimensional coordinates reconstructed by FLAMINGO and the actual Hi-C interactions in different regions of GM12878 cell line (GSE63525) (chr21:31375000-32985000, chr21:30125000-30915000). (**C, D**) RMSD and Coordinate error are employed to quantify the differences between the reconstructed three-dimensional chromatin structures using various methods and the simulate structure. (**E**) Coordinate error are employed to quantify the differences between the high-resolution reconstructed three-dimensional chromatin structures methods at different lengths of chromatin segments loci. (**F, G**) Ratio of nodes occupying Loop anchors in three patterns connected to DSBs in NHEK and K562 cell lines.

FigS10:

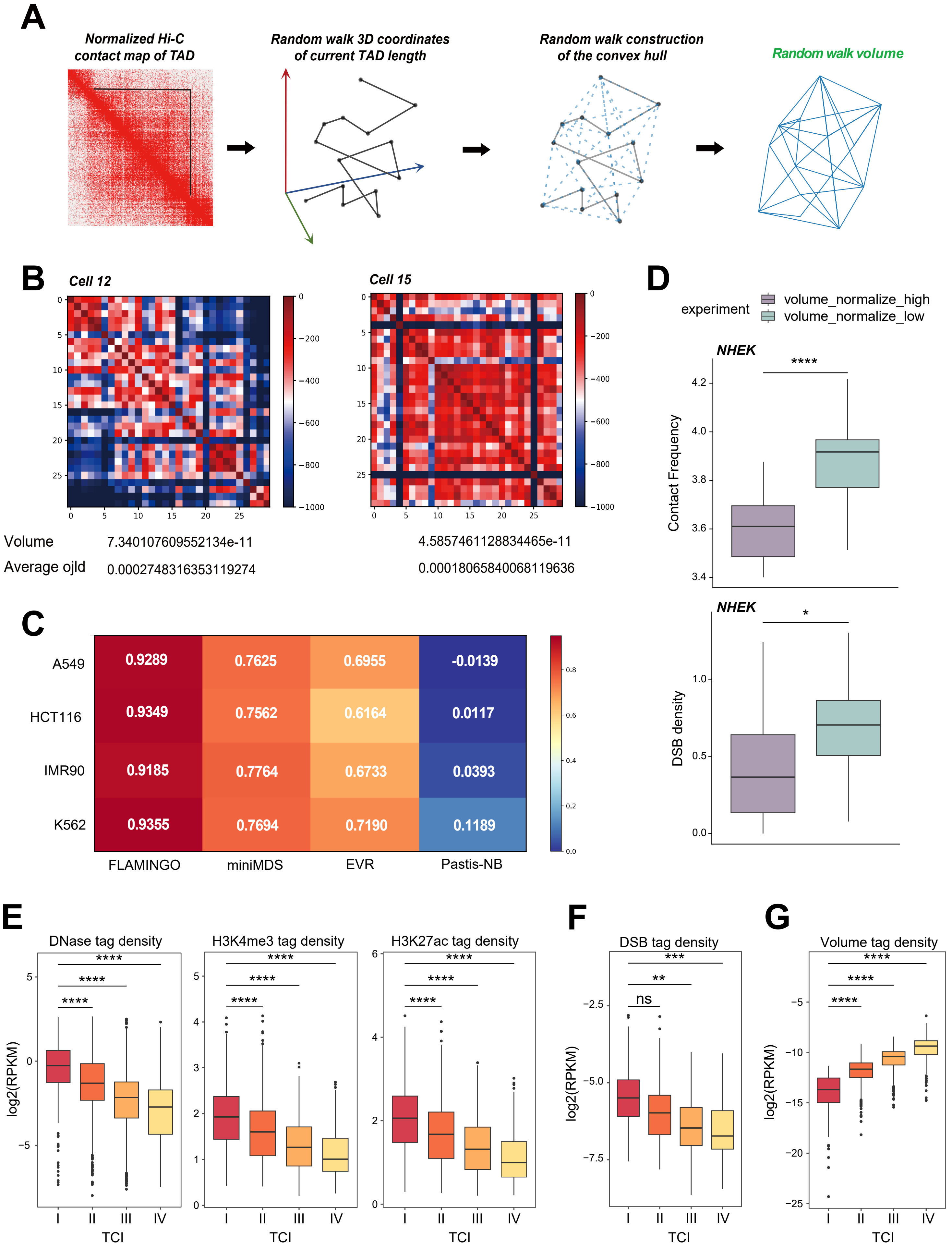

A
Normalized Hi-C contact map of TAD
Random walk 3D coordinates of current TAD length
Random walk construction of the convex hull
Random walk volume
B
Cell 12
Cell 15
Volume
7.340107609552134e-11
4.5857461128834465e-11
Average ojld
0.0002748316353119274
0.00018065840068119636
C
A549
HCT116
IMR90
K562
0.9289
0.7625
0.6955
-0.0139
0.9349
0.7562
0.6164
0.0117
0.9185
0.7764
0.6733
0.0393
0.9355
0.7694
0.7190
0.1189
FLAMINGO
miniMDS
EVR
Pastis-NB
D
experiment
volume_normalize_high
volume_normalize_low
NHEK
****
Contact Frequency
NHEK
*
DSB density
E
DNase tag density
H3K4me3 tag density
H3K27ac tag density
log2(RPKM)
TCI
F
DSB tag density
***
**
ns
G
Volume tag density
I
II
III
IV

Figure S10. **Performance and the reliability of TCI.** (**A**) Schematic diagram of current TAD random walk volume calculation (see methods). (**B**) The two single-cell at the same chromatin fragment regions (IMR90, chr21:31.85-33.35 Mb) with identical genomic coordinates occupying different volumes and average Euclidean distances in physical space. (**C**) Correlation between volumes obtained from reconstruction methods and image tracking. (**D**) With TAD length held constant, the differences in TAD volume reflect the variations in Interactions and DSB density. (**E**) Epigenetic signature for the four groups of TADs in K562 (I-IV, divided by quantile-normalized TCI from low to high). **P < 0.01, ***P < 0.001, ****P < 0.0001, Wilcox test. (**F**) Comparison of DSB density for four groups of TADs in K562 (I-IV, divided by quantile-normalized TCI from low to high) ****P <0.0001, Wilcox test. (**G**) Comparison of TAD volume for four groups of TADs in K562 (I-IV, divided by quantile-normalized TCI from low to high) ****P <0.0001, Wilcox test.

FigS11:

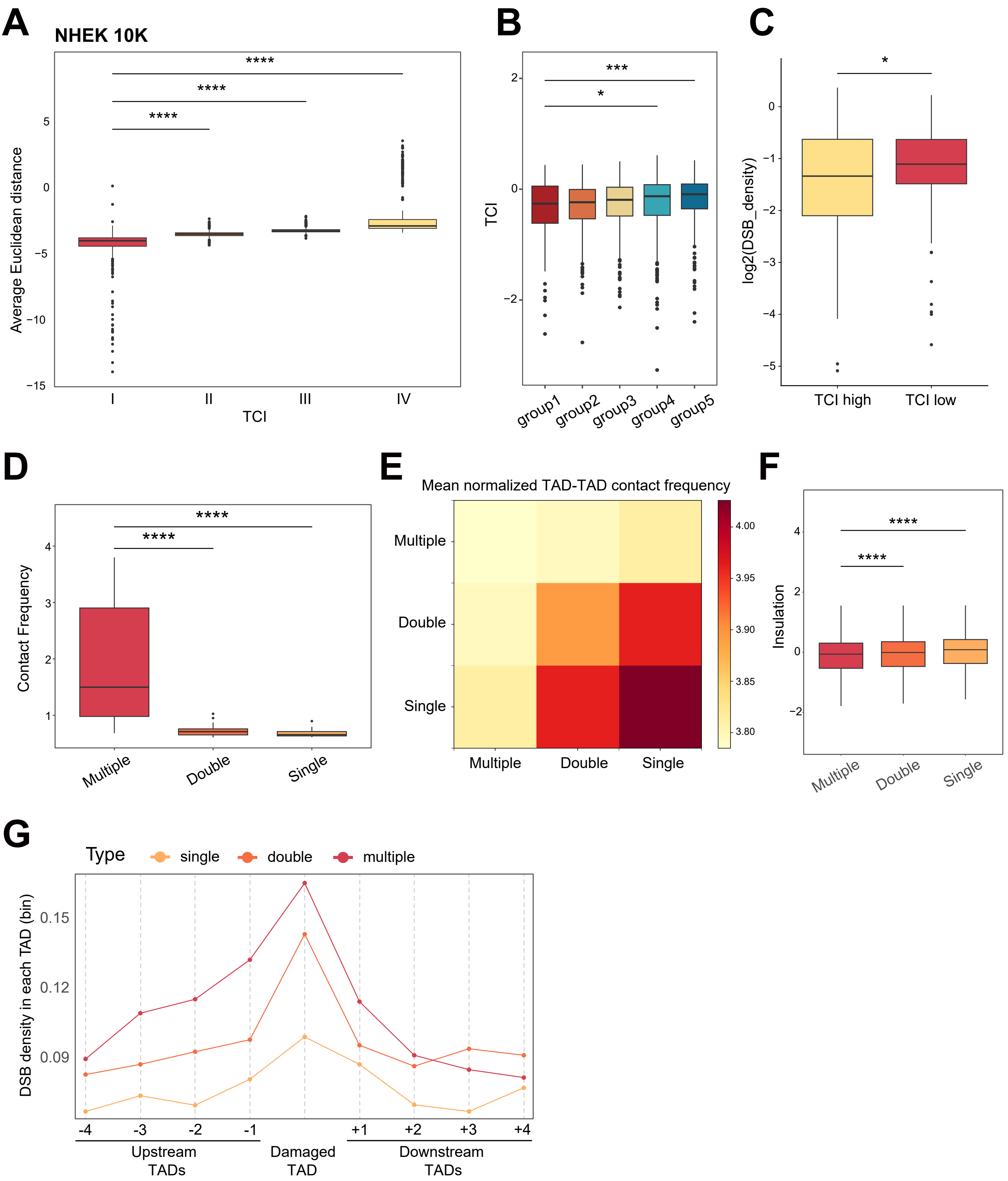
A
NHEK 10K
Average Euclidean distance
TCI
I
II
III
IV
B
TCI
group1
group2
group3
group4
group5
C
log2(DSB_density)
TCI high
TCI low
D
Contact Frequency
Multiple
Double
Single
E
Mean normalized TAD-TAD contact frequency
Multiple
Double
Single
F
Insulation
Multiple
Double
Single
G
Type
single
double
multiple
DSB density in each TAD (bin)
Upstream TADs
Damaged TAD
Downstream TADs

Figure S11. **Chromatin organization and TAD features are associated with DSB distribution patterns.** (**A**) Comparison of average Euclidean distance for four groups of TADs (I-IV, divided by quantile-normalized TCI from low to high) ****P <0.0001, Wilcox test. (**B**) TCI of TADs in each cluster. (**C**) With TAD length held constant, the differences in TCI reflect the variations in DSB density. (**D**) Differences in TAD internal interactions connected to DSBs across three patterns. **P < 0.01, ***P < 0.001, ****P < 0.0001, Wilcox test. (**E**) Heatmap showing mean normalized TAD-TAD contact frequency for the three types of TADs in K562 cell line. (**F**) Differences in insulation among TAD boundaries under different patterns in K562 cell line. ****P <0.0001, Wilcox test. (**G**) Line-chart showing different DSB density within the damaged TAD and adjacent TAD.

FigS12:

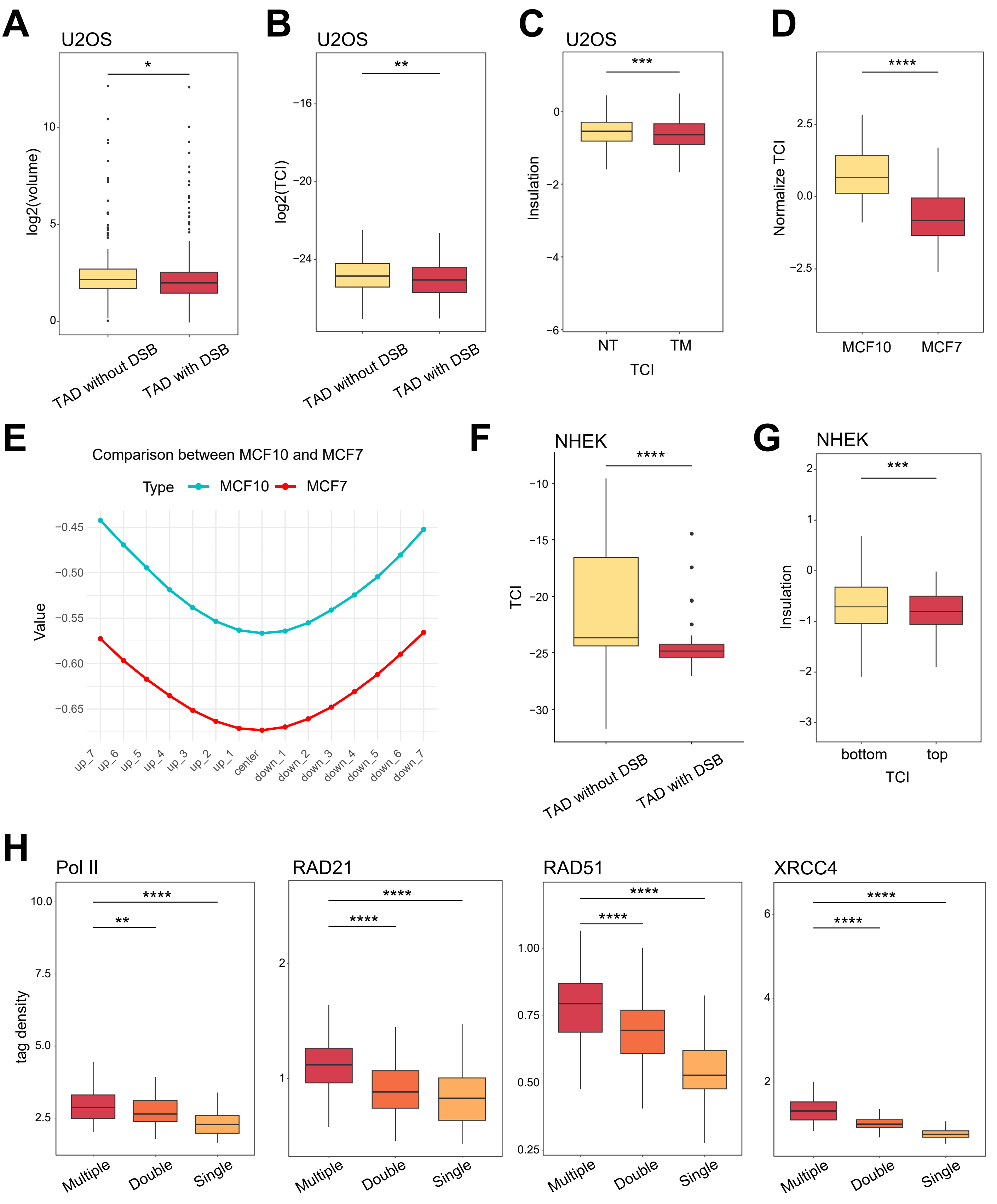
A
U2OS
log2(volume)
TAD without DSB
TAD with DSB
B
U2OS
log2(TCI)
C
U2OS
Insulation
NT
TM
TCI
D
Normalize TCI
MCF10
MCF7
E
Comparison between MCF10 and MCF7
Type MCF10 MCF7
Value
F
NHEK
TCI
G
NHEK
Insulation
bottom
top
TCI
H
Pol II
RAD21
RAD51
XRCC4
tag density
Multiple
Double
Single

Figure S12. **Chromatin organization and TAD features are associated with DSB distribution patterns.** (**A**) Volume differences between two classes of TADs before and after OHT induction in U2OS cells, with TAD length held constant. **P* < 0.05, ***P* < 0.01, ****P* < 0.001, *****P* < 0.0001, Wilcox test. (**B**) TCI differences between two classes of TADs before and after OHT induction in U2OS cells, with TAD length held constant. **P* < 0.05, ***P* < 0.01, ****P* < 0.001, *****P* < 0.0001, Wilcox test. (**C**) Boundary insulation differences between two classes of TADs before and after OHT induction in U2OS cells, with TAD length held constant. **P* < 0.05, ***P* < 0.01, ****P* < 0.001, *****P* < 0.0001, Wilcox test. (**D**) TCI differences between TADs with and without DSBs during the transition from MCF10 to MCF7 cells. **P* < 0.05, ***P* < 0.01, ****P* < 0.001, *****P* < 0.0001, Wilcox test. (**E**) Line chart showing boundary insulation levels within the center TAD and its adjacent TAD. (**F**) TCI differences between TADs with and without DSBs in NHEK cells, with TAD length held constant. **P* < 0.05, ***P* < 0.01, ****P* < 0.001, *****P* < 0.0001, Wilcox test. (**G**) Boundary insulation differences between TADs with and without DSBs in NHEK cells, with TAD length held constant. **P* < 0.05, ***P* < 0.01, ****P* < 0.001, *****P* < 0.0001, Wilcox test. (**H**) Enrichment of DNA damage repair signals in three types of TADs, including Pol II in NHEK cells and RAD21, RAD51, and XRCC4 in K562 cells. **P* < 0.05, ***P* < 0.01, ****P* < 0.001, *****P* < 0.0001, Wilcox test.

FigS13:

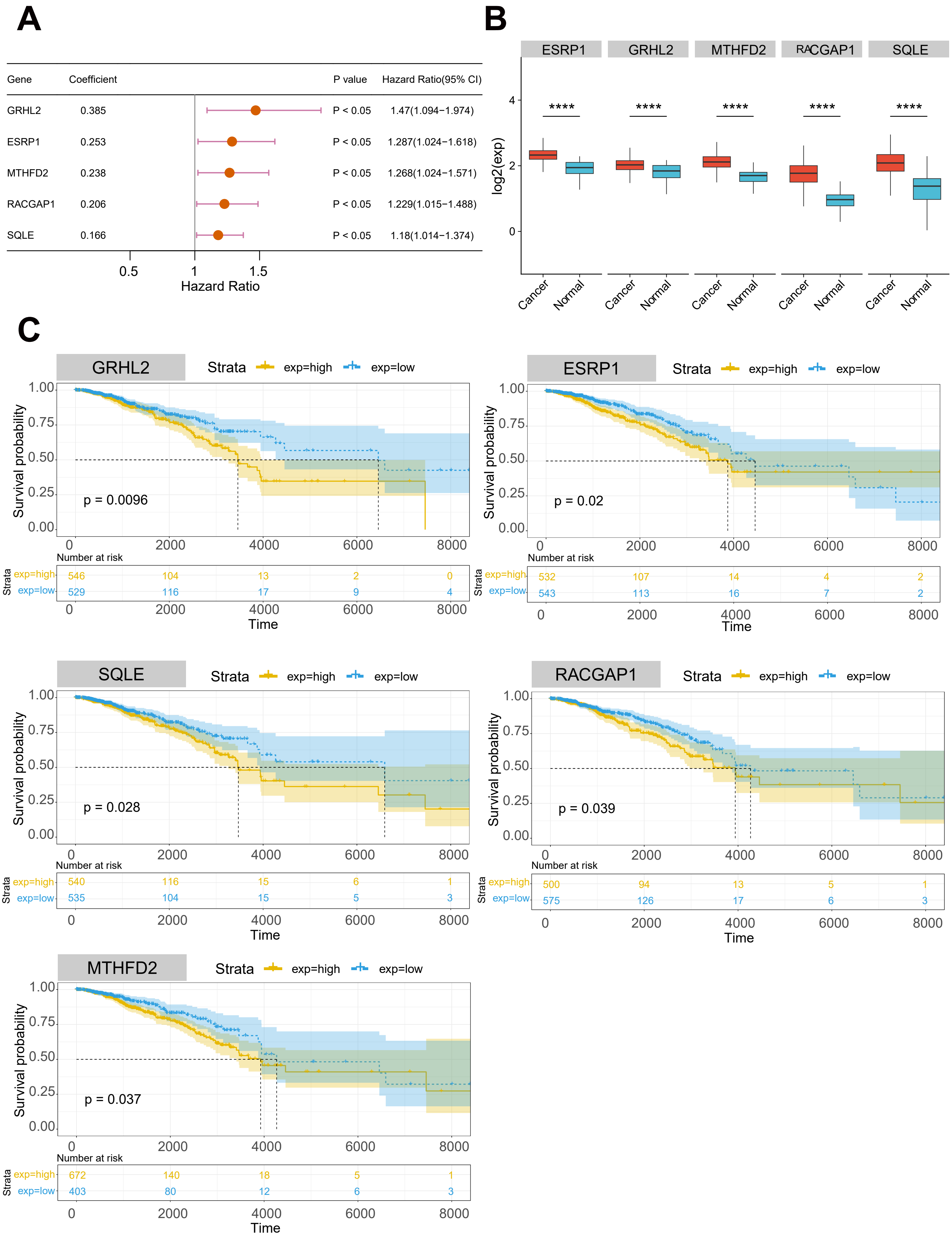

A
Gene
Coefficient
P value
Hazard Ratio(95% CI)
GRHL2
0.385
P < 0.05
1.47(1.094−1.974)
ESRP1
0.253
P < 0.05
1.287(1.024−1.618)
MTHFD2
0.238
P < 0.05
1.268(1.024−1.571)
RACGAP1
0.206
P < 0.05
1.229(1.015−1.488)
SQLE
0.166
P < 0.05
1.18(1.014−1.374)
0.5
1
1.5
Hazard Ratio
B
ESRP1
GRHL2
MTHFD2
RACGAP1
SQLE
****
log2(exp)
4
2
0
Cancer
Normal
C
GRHL2
Strata
exp=high
exp=low
Survival probability
1.00
0.75
0.50
0.25
0.00
p = 0.0096
0
2000
4000
6000
8000
Number at risk
Strata
546
104
13
2
0
529
116
17
9
4
Time
ESRP1
p = 0.02
532
107
14
4
2
543
113
16
7
2
SQLE
p = 0.028
540
116
15
6
1
535
104
15
5
3
RACGAP1
p = 0.039
500
94
13
5
1
575
126
17
6
3
MTHFD2
p = 0.037
672
140
18
5
1
403
80
12
6
3

Figure S13. **Screening for genes associated with breast cancer survival.** (**A**)Forest maps of oncogenes and suppressor genes related to prognosis of breast cancer was analyzed by univariate COX. (**B**) Boxplots showing the transcription levels of our screened oncogenes and suppressor genes in cancer and normal tissues, respectively. *P < 0.05, **P < 0.01, ***P < 0.001, ****P < 0.0001, Wilcox test.(**C**) Kaplan-Meier curves displaying relapse-free survival for patients with breast cancer based on gene expression. High expression of genes shown in yellow and low expression is shown in blue. p value was derived from the log rank test.

FigS14:

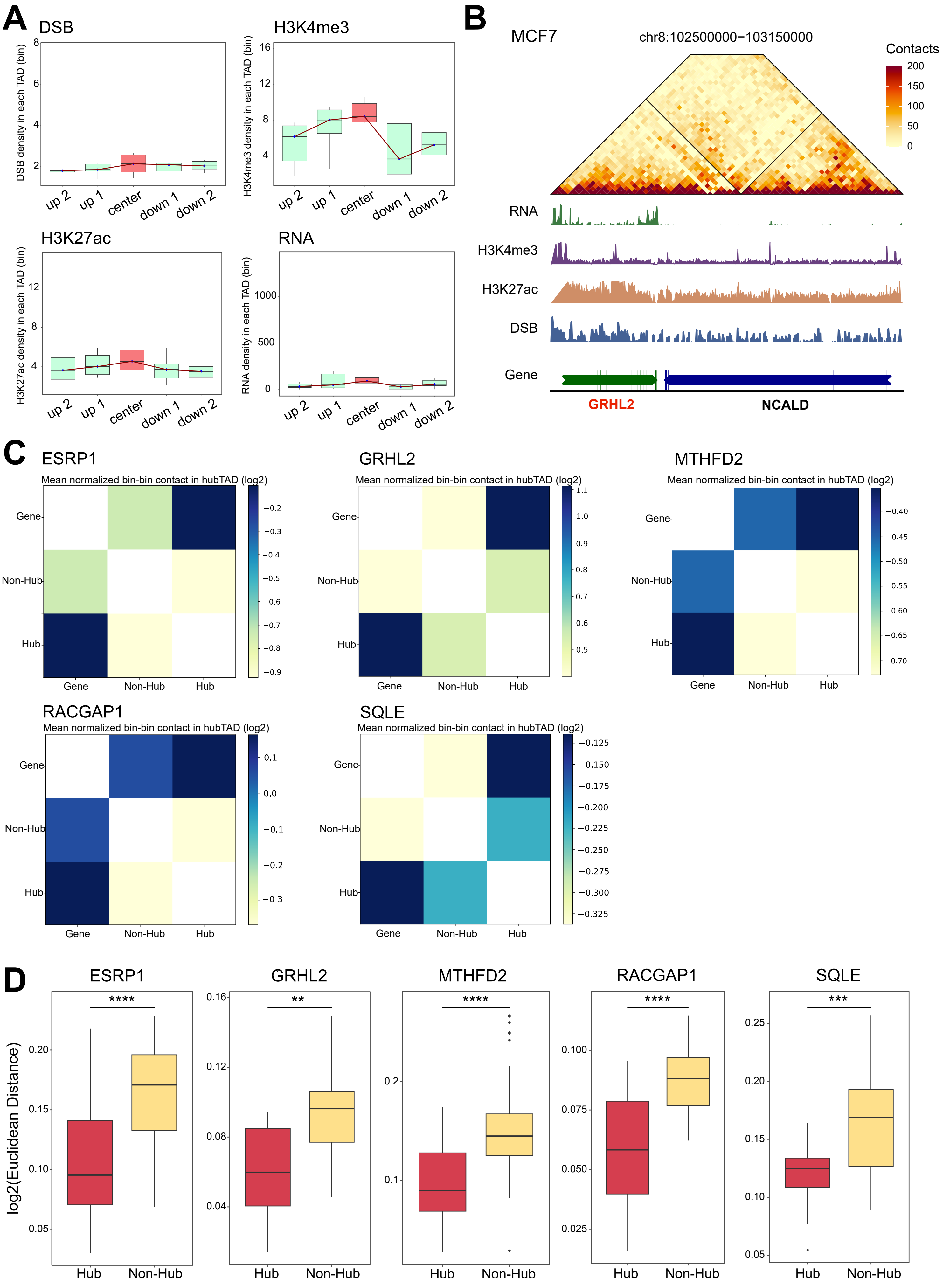
A
DSB
H3K4me3
H3K27ac
RNA
DSB density in each TAD (bin)
H3K4me3 density in each TAD (bin)
H3K27ac density in each TAD (bin)
RNA density in each TAD (bin)
up 2
up 1
center
down 1
down 2
B
MCF7
chr8:102500000−103150000
Contacts
200
150
100
50
0
RNA
H3K4me3
H3K27ac
DSB
Gene
GRHL2
NCALD
C
ESRP1
GRHL2
MTHFD2
RACGAP1
SQLE
Mean normalized bin-bin contact in hubTAD (log2)
Gene
Non-Hub
Hub
D
ESRP1
GRHL2
MTHFD2
RACGAP1
SQLE
log2(Euclidean Distance)
****
**
****
****
***
Hub
Non-Hub

Figure S14. **Screening for genes associated with breast cancer survival.** (**A**) The box plot illustrates the enrichment of DSBs, H3K4me3, H3K27ac, and RNA levels between the TADs containing the five risk genes and their surrounding TADs. (**B**) Top: An example Hi-C heatmap of a region (chr8:102500000-103150000) in MCF7. Middle: An illustration of TADs containing risk gene (*GRHL2*). Bottom: RNA, H3K27ac, H3K4me3, and DSB data on the specified genomic region. (**C**) Heatmap showing the interaction patterns among risk nodes, hub nodes, and non-hub nodes, excluding self-interactions along the diagonal. Rows and columns: Represent different types of nodes (risk nodes, hub nodes, and non-hub nodes). Color Scale: Indicates the level of interaction frequency, with warmer colors representing lower interaction frequencies and cooler colors representing higher interaction frequencies. (**C**) Box plot illustrating the 3D Euclidean distances between the bin containing risk genes and both hub nodes and non-hub nodes. *P < 0.05, **P < 0.01, ***P < 0.001, ****P < 0.0001, Wilcox test.
