# Supplementary material for: ChromInSight: Revealing DNA Double‐Strand Breaks Through Chromatin Structural Insights With an Interpretable Graph Neural Network Framework
Source: Adv Sci (Weinh). 2025 Jun 30;12(36):e04571. doi: 10.1002/advs.202504571 (PMC12463085; doi:10.1002/advs.202504571)
Supplement: Supplementary file 2 — Supporting Information [file ADVS-12-e04571-s002.pdf]

ADVANCED SCIENCE Open Access

Supporting Information

for *Adv. Sci.*, DOI 10.1002/advs.202504571

ChromInSight: Revealing DNA Double-Strand Breaks Through Chromatin Structural Insights With an Interpretable Graph Neural Network Framework

Kang Xu, Zongyuan Yu, Canzhuang Sun, Conglin Gou, Jiangyue Zhu, Jun Wang, Xiaochen Bo, Guoxian Yu*, Hao Li* and Hebing Chen**

Supporting Information

TableS1：Software and parameter selection for calling peaks in different articles

Author	Journal	PMID	Software	Method	Parameters
Andre´ Nussenzweig	Cell	28725753	MACS 1.4.3	End-seq	--nolambda<br>--nomodel<br>--keep-dup=all
Andre´ Nussenzweig	Mol Cell	27477910	HOMER v4.8.2<br>SICER v1.1	End-seq	-regionsize 150<br>-minDist 500<br>-minreads 35
Stefanie V. Lensing	Nat Methods	27525976	MACS2	DSBCapture	--nomodel<br>-q 0.05
Stefanie V. Lensing	Nat Methods	27525976	MACS2	BLESS	--nomodel<br>-q 0.05
Winston X. Yan	Nat Commum	28497783	Custom Scripts	BLISS	

TableS2：Statistical Analysis of Model Performance.

A. The average AUROC ± standard deviation. (20 replicates)

Method	AUROC±std	AUPRC±std
Hi-DSB	0.9344 ± 0.000113	0.8226 ± 0.000252
DSB-GNN	0.9115 ± 0.000521	0.7590 ± 0.001152
RF	0.8769 ± 0.000816	0.7023 ± 0.001913

B. DeLong test for model statistical comparison

Method1	Method2	DeLong(P-Value)
Hi-DSB	DSB-GNN	1.486e-168
Hi-DSB	RF	2.442e-161
DSB-GNN	RF	2.115e-31

C. McNemar test for model statistical comparison

Method1	Method2	McNemar(P-Value)
Hi-DSB	DSB-GNN	6.814e-143
Hi-DSB	RF	2.478e-146
DSB-GNN	RF	8.925e-02

TableS3: Model performance was evaluated using AUROC.

Normalization **RAW**		10-kb			25-kb			50-kb		
		**Hi-DSB**	**DSB-GNN**	**RF**	**Hi-DSB**	**DSB-GNN**	**RF**	**Hi-DSB**	**DSB-GNN**	**RF**
Cell line	NHEK	0.93354	0.91181	0.88064	0.93917	0.91595	0.89733	0.95089	0.92984	0.92000
	K562	0.84782	0.79978	0.83510	0.89568	0.88699	0.88699	0.92423	0.91831	0.91831
	MCF7	0.88737	0.87857	0.87857	0.91720	0.91062	0.91062	0.92995	0.92452	0.92452

Normalization **OE**		10-kb			25-kb			50-kb		
		**Hi-DSB**	**DSB-GNN**	**RF**	**Hi-DSB**	**DSB-GNN**	**RF**	**Hi-DSB**	**DSB-GNN**	**RF**
Cell line	NHEK	0.93411	0.91104	0.88136	0.93941	0.91557	0.89781	0.95109	0.93122	0.91898
	K562	0.84625	0.81065	0.83357	0.89163	0.88263	0.88263	0.91787	0.91185	0.91185
	MCF7	0.88432	0.87471	0.87471	0.91429	0.90656	0.90656	0.92546	0.91922	0.91922

Normalization **KR**		10-kb			25-kb			50-kb		
		**Hi-DSB**	**DSB-GNN**	**RF**	**Hi-DSB**	**DSB-GNN**	**RF**	**Hi-DSB**	**DSB-GNN**	**RF**
Cell line	NHEK	0.93272	0.91181	0.88208	0.93825	0.91581	0.89796	0.95002	0.92965	0.91687
	K562	0.84169	0.79978	0.82852	0.88702	0.87637	0.87637	0.91425	0.90772	0.90772
	MCF7	0.88248	0.87857	0.87140	0.91238	0.90474	0.90474	0.92306	0.91630	0.91630

TableS4: Model performance was evaluated using AUPRC.

Normalization **RAW**		10-kb			25-kb			50-kb		
		**Hi-DSB**	**DSB-GNN**	**RF**	**Hi-DSB**	**DSB-GNN**	**RF**	**Hi-DSB**	**DSB-GNN**	**RF**
Cell line	NHEK	0.82192	0.75709	0.71120	0.91667	0.88007	0.85289	0.96058	0.94214	0.92689
	K562	0.83611	0.77795	0.81696	0.86578	0.80077	0.85048	0.90465	0.84407	0.89386
	MCF7	0.85017	0.76039	0.83739	0.90458	0.81093	0.89533	0.91034	0.83194	0.90209
Normalization **OE**		10-kb			25-kb			50-kb		
		**Hi-DSB**	**DSB-GNN**	**RF**	**Hi-DSB**	**DSB-GNN**	**RF**	**Hi-DSB**	**DSB-GNN**	**RF**
Cell line	NHEK	0.82163	0.75599	0.71016	0.91645	0.87991	0.85088	0.96037	0.94293	0.92668
	K562	0.83224	0.79334	0.81400	0.85853	0.79863	0.84257	0.89736	0.83229	0.88627
	MCF7	0.84335	0.76728	0.82932	0.89854	0.82193	0.88786	0.90437	0.81808	0.89591
Normalization **KR**		10-kb			25-kb			50-kb		
		**Hi-DSB**	**DSB-GNN**	**RF**	**Hi-DSB**	**DSB-GNN**	**RF**	**Hi-DSB**	**DSB-GNN**	**RF**
Cell line	NHEK	0.82100	0.75778	0.71240	0.91614	0.88038	0.85259	0.95990	0.94216	0.92523
	K562	0.82503	0.77584	0.80640	0.85034	0.79108	0.83286	0.88934	0.83522	0.87841
	MCF7	0.83928	0.76605	0.82427	0.89354	0.81260	0.88204	0.89833	0.80999	0.88638

TableS5: Ablation results for removing each single component of Hi-DSB

Information Type	Specific Setting	AUC
node sub-feature	CTCF+DNase+H3K4me3+H3K27ac (Hi-DSB)	0.9342
	CTCF+DNase+H3K4me3	0.9238
	CTCF+DNase+H3K27ac	0.9290
	CTCF+H3K4me3+H3K27ac	0.9141
	DNase+H3K4me3+H3K27ac	0.9221
	DNase+H3K4me3	0.9129
	DNase+H3K27ac	0.9155
	H3K4me3+H3K27ac	0.8850
	CTCF	0.8074
	DNase	0.8423
Hi-C network	with real Hi-C network (Hi-DSB)	0.9342
	with Hi-C down-sample 20%	0.9286
	with Hi-C down-sample 40%	0.9173
	with Hi-C down-sample 60%	0.8962
	with Hi-C down-sample 80%	0.8753
Model components	with self-attention (Hi-DSB)	0.9342
	without self-attention	0.9015
	without graph contrastive learning(GCL)	0.8793
	without JK structure	0.8851

TableS6：Statistical Analysis of Model Performance in HCT116.

A. DeLong test for model statistical comparison

Method1	Method2	DeLong(P-Value)
Hi-DSB	DSB-GNN	1.339e-30
Hi-DSB	RF	2.878e-10
DSB-GNN	RF	2.103e-09

B. McNemar test for model statistical comparison

Method1	Method2	McNemar(P-Value)
Hi-DSB	DSB-GNN	2.032e-12
Hi-DSB	RF	1.546e-05
DSB-GNN	RF	1.498e-21

TableS7: **siRNA-mediated knockdown in MCF7.**

A. The efficiency of siRNA-mediated gene knockdown using qPCR

siRNA	Primer	Reapeat 1	Reapeat 2	Reapeat 3	Reapeat 4
NC	GAPDH	18.53	19.20	18.49	19.28
ESRP1	GAPDH	18.98	18.53	19.07	19.65
GRHL2	GAPDH	18.84	17.87	17.87	18.07
MTHFD2	GAPDH	23.78	22.31	19.45	20.58
RACGAP1	GAPDH	19.57	19.44	19.65	19.43
SQLE	GAPDH	18.6	18.2	18.25	18.33
NC	ESRP1	21.13	22.51	21.30	23.28
NC	GRHL2	21.02	22.50	21.22	22.79
NC	MTHFD2	20.9	22.28	20.83	22.39
NC	RACGAP1	20.55	22.04	20.87	22.78
NC	SQLE	31.96	34.52	30.41	30.52
ESRP1	ESRP1	25.69	25.18	25.38	25.18
GRHL2	GRHL2	26.6	25.15	25.46	25.38
MTHFD2	MTHFD2	26.11	24.61	25.85	25.85
RACGAP1	RACGAP1	26.06	23.75	23.73	24.39
SQLE	SQLE	32.89	33.93	38.32	34.96

B. Cell counts under siRNA interference

repeat	negative control	ESRP1	GRHL2	MTHFD2	RACGAP1	SQLE
1	1.98E+05	8.72E+04	1.67E+05	1.49E+05	6.00E+04	1.39E+05
2	1.81E+05	7.49E+04	1.44E+05	1.16E+05	5.72E+04	1.24E+05
3	1.81E+05	1.26E+05	1.15E+05	1.05E+05	6.41E+04	1.01E+05
4	1.36E+05	1.02E+05	1.22E+05	1.48E+04	7.49E+04	1.20E+05

TableS8: **Primer sequence.**

A.SiRNA Sequence List

name	sequence
hESRP1-s	GGUAUAUUGAGGUUUACAA/dT//dT/
hESRP1-a	UUGUAAACCUCAAUAUACC/dT//dT/
hGRHL2-s	GCGAGACCGGAGACAACAA/dT//dT/
hGRHL2-a	UUGUUGUCUCCGGUCUCGC/dT//dT/
hMTHFD2-s	GGAUCAGUAUUCCAUGUUA/dT//dT/
hMTHFD2-a	UAACAUGGAAUACUGAUCC/dT//dT/
hRACGAP1-s	AGGUGGAUGUAGAGAUCAA/dT//dT/
hRACGAP1-a	UUGAUCUCUACAUCCACCU/dT//dT/
hSQLE-s	CCCAGGUUGUAAAUGGUUA/dT//dT/
hSQLE-a	UAACCAUUUACAACCUGGG/dT//dT/

B.DNA Primer Sequence List

name	sequence
hESRP1-f	ATCCTGGATTTCCTGGGGGA
hESRP1-r	CATGGCGGTGGGGATAAGC
GAPDH-1.r	GTCGGAGTCAACGGATTTGG
GAPDH-1.f	GATTCCACCCATGGCAAATTC
hGRHL2-f	GGGGGACGGAAAAGCAGAAT
hGRHL2-r	CACTGGTGTAGGCTCTTCGG
hMTHFD2-f	GTGATCCTGGTTGGCGAGAA
hMTHFD2-r	TGCTCTGGAAGAGGCAACTG
hRACGAP1-f	TTGACGTTGAATAGGGAGAGCC
hRACGAP1-r	ATCCATCTTTCCTAAGTGCTACAG
hSQLE-f	GCCTGCCTTTCATTGGCTTC
hSQLE-r	TTCCTTTTCTGCGCCTCCTG

TableS9：DSB dataset overview

Cell line	Process	Source	Accession
NHEK	No treatment	DSBCapture	GSE78172
NHEK	No treatment	BLESS	GSE78172
K562	No treatment	BLISS	SRP150602
K562	Etoposide	BLISS	SRP150602
MCF7	No treatment	BLISS	SRP150602
Nalm6	No treatment	END-seq	GSE99197
Nalm6	Etoposide	END-seq	GSE99197
